# Development of recombinant fowl adenovirus serotype 4 harboring the HiBiT-Tag reporter and its utility in antiviral research

**DOI:** 10.1128/spectrum.03347-25

**Published:** 2026-01-28

**Authors:** Xiaoran Guo, Cheng Li, Lingzhai Meng, Xiuli Li, Fuqiang Li, Lili Wang, Zhimin Dong, Qi Zhu, Huizhong Sun, Li Zhang, Minghua Yan

**Affiliations:** 1 Tianjin Academy of Agricultural Sciences, Institute of Animal Science and Veterinary 260607, Tianjin, China; 2 Tianjin Key Laboratory of Animal Molecular Breeding and Biotechnology, Tianjin, China; 3 Tianjin Engineering Research Center of Animal Healthy Farming, Tianjin, China; 4 Tianjin Observation and Experimental Site of National Animal Health, Tianjin, China; 5 College of Animal Science and Veterinary Medicine, Tianjin Agricultural University 91633, Tianjin, China; 6 Tianjin Academy of Agricultural Sciences, Institute of Agro-product Safety and Nutrition 260607, Tianjin, China; The Ohio State University College of Veterinary Medicine, Columbus, Ohio, USA

**Keywords:** fowl adenovirus serotype 4, reverse genetics system, insertion site, HiBiT, antiviral drugs

## Abstract

Severe hydropericardium-hepatitis syndrome (HHS) caused by fowl adenovirus 4 (FAdV-4) significantly affects the global poultry industry. However, there is currently no convenient and sensitive platform available for the rapid screening of antiviral drugs and the detection of neutralizing antibodies. Here, we first established an improved reverse genetics system for FAdV-4 Y17215-1 strain and identified the optimal insertion site for expressing foreign genes (between the ORF19A and ORF4 genes). To obtain a quantifiable recombinant reporter FAdV-4, the HiBiT-tag reporter gene was inserted into this site, and recombinant FAdV-4 expressing the HiBiT gene (rFAdV-4-HiBiT) was successfully rescued. Further analysis showed that rFAdV-4-HiBiT had similar growth kinetics to the parental virus and retained luciferase activity and genetic stability after 10 rounds of serial passages. A proof-of-concept test confirmed that rFAdV-4-HiBiT can be used to rapidly quantify anti-FAdV-4 neutralizing antibodies in chicken serum samples and screen for antiviral agents, including antiviral drugs and proteins, based on the intensity of luciferase activity. Collectively, the HiBiT-tagged virus rFAdV-4-HiBiT provides a robust tool for the rapid detection of FAdV-4 and facilitates the development of novel therapeutics and vaccines for FAdV-4.

**IMPORTANCE:**

The epidemic spread of fowl adenovirus 4 (FAdV-4) presents significant challenges for the global poultry industry. However, there are currently few convenient and sensitive platforms available for antiviral drug screening and neutralizing antibody detection. In this study, we first established an improved reverse genetics system for the FAdV-4 and screened for the optimal insertion site for foreign genes between ORF19A and ORF4. Furthermore, the HiBiT gene was further inserted into this site, and rFAdV-4-HiBiT was successfully rescued. A luciferase-based FAdV-4 neutralizing antibody detection method has been successfully established, which can reduce detection time and greatly enhance the efficiency of neutralizing antibody testing. Furthermore, this system can serve as a more convenient screening platform for anti-FAdV-4 drugs. Collectively, rFAdV-4-HibiT represents an important tool with great potential for facilitating the development of novel therapeutics and vaccines for FAdV-4.

## INTRODUCTION

Fowl adenovirus (FAdV) infections are commonly associated with several important poultry diseases, such as hydropericardium-hepatitis syndrome (HHS) (1, 2), inclusion body hepatitis (3, 4) and gizzard erosion (5, 6), which cause severe economic losses worldwide. Recently, the International Committee on Taxonomy of Viruses classification system divided FAdVs into 5 species and 12 serotypes, designated FAdV-1 to -7, -8a, -8b, and -9 to -11 (7). FAdV-4 is particularly important among the 12 serotypes due to its high lethality and widespread prevalence (8). FAdV-4 is a member of *Adenoviridae*, *avian adenovirus* family and contains a linear double-stranded DNA genome of approximately 45 kb in length, encoding 11 structural and 32 non-structural proteins (9, 10). Hexon and fiber 2 are the two main viral capsid proteins closely related to the virulence of FAdV-4 (11–13).

Since 2015, FAdV-4 infections and outbreaks caused by a highly virulent novel strain have become widespread in poultry production regions of China (14). The FAdV-4-infected chickens typically present with HHS pathologic lesions and mortality rates of approximately 100% (15–18). Sun et al. used the FAdV-4 (SDSG) strain to orally and intramuscularly inoculate 10-day-old and 20-day-old SPF chickens for the evaluation of pathogenicity. The results demonstrated that typical hydropericardium and hepatitis were observed in the infected chickens (19). Yuan et al. used the hypervirulent FAdV-4 to inoculate 7- to 180-day-old SPF chickens intramuscularly, and the results showed that the incidence rate and mortality of chickens under 59 days old were 100% (20). Although various types of FAdV-4 vaccines have been developed to prevent and control HHS (21), there are virtually no antiviral drugs effective against FAdV-4. Therefore, there is an urgent need for a rapid screening platform to develop antiviral drugs.

Recombinant viruses expressing specific reporter genes have been widely applied in antiviral research and neutralizing antibody assays (22, 23). For instance, the recombinant Semliki Forest virus (SFV) expressing the EGFP reporter gene (SFV-EGFP) enables rapid screening of potent broad-spectrum inhibitors of alphaviruses (24). In a high-throughput neutralizing antibody assay based on recombinant avian influenza virus (AIV), an AIV-NanoLuciferase (AIV-Nluc) provided 20-fold higher sensitivity than that of the traditional cytopathic effect (CPE) neutralizing antibody assay (25). However, because of their large size, inserting EGFP or Nluc into specific viral genomes may result in reduced replication levels and rescue failure of recombinant virus (26). The small peptide tag HiBiT, including a smaller size (an 11-amino-acid peptide), has a strong affinity for a larger subunit called LgBiT (158-amino-acid peptide), and the combination of these two components forms a catalytically active NanoLuc luciferase complex, which generates a bright luminescent signal. Thus, HiBiT-labeled proteins can be detected and quantified using the Nano-Glo assay system (27). Compared to firefly and Renilla luciferases, the HiBiT tag presents several advantages, including a smaller size, high stability, better sensitivity, and dynamic range. Currently, HiBiT has been successfully used in various reporter viruses, including severe acute respiratory syndrome coronavirus 2 (28), porcine reproductive and respiratory syndrome virus (29), bovine rotavirus (30), hepatitis E virus (HEV) (31), and enterovirus A71 (26). These studies have collectively demonstrated the suitability and genetic stability of HiBiT.

Although a visualized recombinant FAdV-4 has been constructed (32), a platform for rapid detection of FAdV-4 neutralizing antibodies and antiviral drug screening remains to be developed because of the non-quantifiable properties of EGFP. Therefore, in this study, we aimed to develop a convenient and sensitive platform for the rapid detection of neutralizing antibodies and screening of antiviral drugs against FAdV-4. We first established an improved FAdV-4 reverse genetics system and identified the optimal insertion site (between ORF19A and ORF4) to be suitable for foreign gene expression. Furthermore, the rescued recombinant FAdV-4 expressing HiBiT gene (rFAdV-4-HiBiT) could be used for rapid detection of neutralizing antibodies and screening of antiviral drugs and proteins.

## MATERIALS AND METHODS

### Virus, cell, and antibodies

The FAdV-4 strain Y17215-1 (GenBank accession number: PQ539419) was isolated from a chicken flock with HHS symptoms at our laboratory (33). LMH cells were cultured in 10% fetal bovine serum (FBS) growth medium of DMEM/F12 at 37°C with 5% CO_2_. Rabbit anti-FAdV-4 fiber 1 polyclonal antibody was stored in our laboratory. Mouse anti-EGFP monoclonal antibodies were obtained from Abcam Group. Mouse monoclonal antibody anti-HA and anti-β-actin were obtained from the Proteintech Group.

### Plasmids, chicken IFN-α (chIFN-α) protein, and drugs

Total RNA of LMH cells was reverse transcribed into cDNA, and the open reading frames of annexin A5 (ANXA5), sequestosome 1 (SQSTM1), heat-shock protein 90 alpha family class A member 1 (HSP90AA1), chaperonin containing TCP1 subunit 5 (CCT5), and MHC class II invariant chain (CD74) genes were amplified from the cDNA. Then, the five amplified fragments were inserted into the pCAGGS-HA vector to construct five plasmids, designated pCAGGS-ANXA5-HA, pCAGGS-SQSTM1-HA, pCAGGS-HSP90AA1-HA, pCAGGS-CCT5-HA, and pCAGGS-CD74-HA. The plasmids (pUC-HiBiT and pEGFP-N1), chIFN-α protein, Honeysuckle flower, and *Ligustrum lucidum* were stored in our laboratory. Ribavirin and apigenin were obtained from Solarbio Group.

### Construction of a reverse genetics system for the FAdV-4

**Fig 1:**
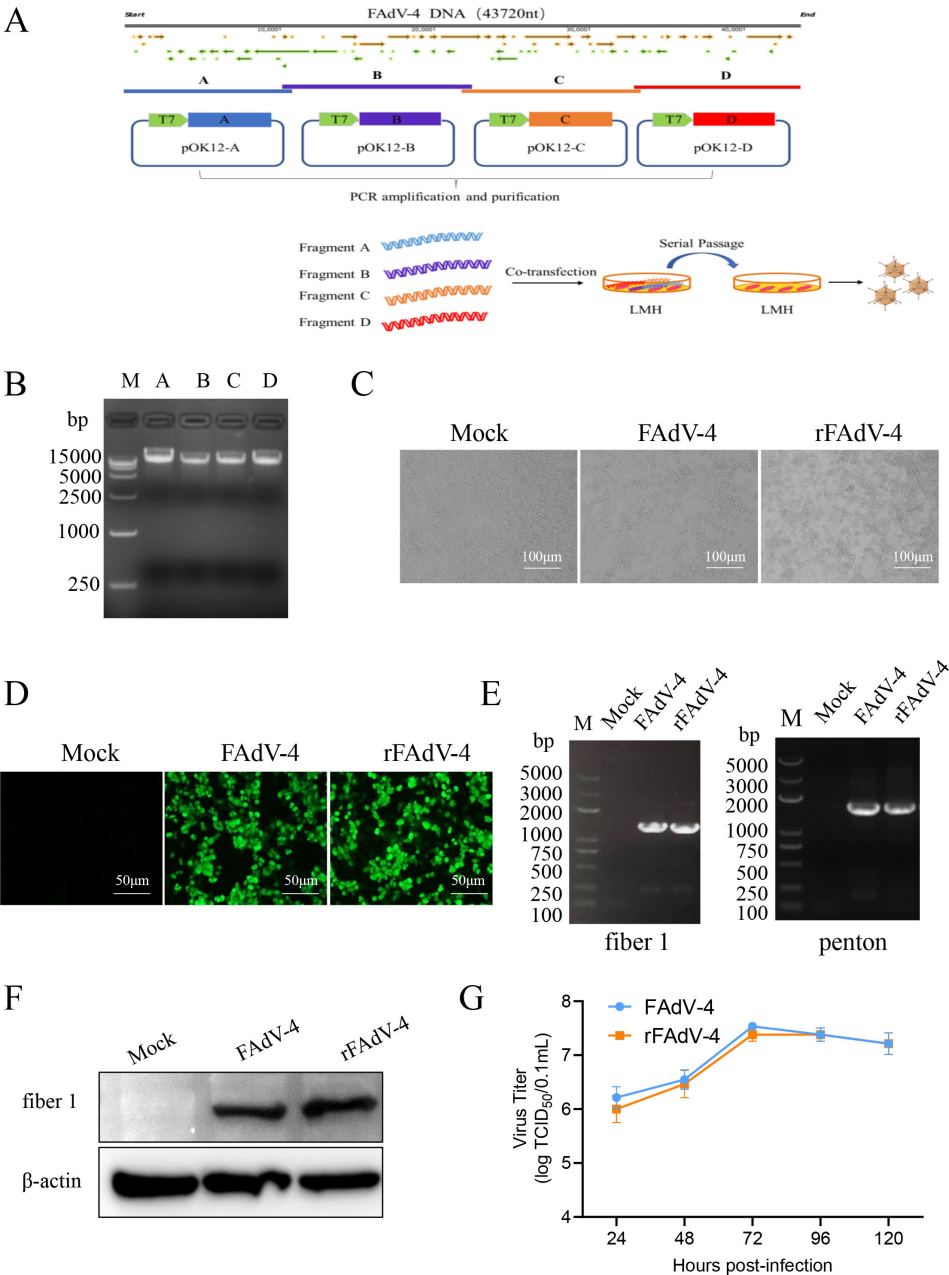
Construction, identification, and characterization of the recombinant fowl adenovirus 4 (rFAdV-4). (**A**) Schematic diagram for the construction of the FAdV-4 reverse genetics system. The complete genome of FAdV-4 was divided into four separate genomic fragments A, B, C, and D, which were then ligated into the pOK12 vector to construct four library plasmids. (**B**) Electrophoresis of the four DNA fragments. Four purified DNA fragments were run on a 1% agarose gel. (**C**) Cytopathic eﬀect (CPE) was observed on the LMH cells infected with the wild-type FAdV-4 and rFAdV-4 after 24 hpi (scale bar, 100 μm). (**D**) Immunofluorescence analysis of the FAdV-4 and rFAdV-4 strains (scale bar, 50 μm). (**E**) Positive PCR bands at 1,296 and 1,577 bp for fiber 1 and Penton genes of FAdV-4 and rFAdV-4 strains. (**F**) Western blotting detected fiber 1 protein of FAdV-4 and rFAdV-4. (**G**) Growth kinetics of the FAdV-4 and rFAdV-4 strains in LMH cells, of which the data were expressed as mean titer and standard deviation of triplicate samples.

To establish a reverse genetics system of the FAdV-4 strain Y17215-1, four separate fragments (A, B, C, and D) were generated using high-fidelity Prime Star GXL DNA polymerase (Takara). Each fragment contained approximately 200 bp homology arms. The total length of the four fragments covered the complete sequence of FAdV-4 Y17215-1. To obtain a linearized vector, pOK12 was digested with *Eco*R I. Subsequently, fragments A, B, C, and D were assembled into pOK12 vector using the NEBuilder HiFi DNA Assembly Cloning Kit (New England BioLabs) and named pOK12-A, pOK12-B, pOK12-C, and pOK12-D, respectively. Subsequently, four plasmids pOK12-A, pOK12-B, pOK12-C, and pOK12-D were used as templates for PCR amplification and purification to obtain four fragments A, B, C, and D covering the full-length genome of FAdV-4. LMH cells were co-transfected with 1 μg each of fragments A, B, C, and D using the X-tremeGENE HP DNA Transfection Reagent (Roche) (Fig. 1A). After 120 h post-transfection, the cells were freeze-thawed twice and passaged continuously via fresh LMH cells. The primers used for clone construction are listed in Table 1.

**TABLE 1:** Primers for the construction and identification of rFAdV-4 infectious clone^*a*^

Name	Primer sequence (5′−3′)
AF	CGTCTGCAGAAGCTTCGAACATCATCTTATATAACCGCGTCTTTTGACAC
AR	TGGTACCCGGGAGCTCGAATCCGATTTCTACTCCGTAGACGTCATCG
BF	CGTCTGCAGAAGCTTCGAAGGTGATTTTGGTTCCACAGTTGTTTTTG
BR	TGGTACCCGGGAGCTCGAAAGGTTTTTGATGGCGAAGAATTTTTGGG
CF	CGTCTGCAGAAGCTTCGAATTACAAGTTTAGCATCTCCGGCTTCGAC
CR	TGGTACCCGGGAGCTCGAATTCATAGTATCCATTGGCCGAGTACGTCC
DF	CGTCTGCAGAAGCTTCGAATACCTCCCTCTACTTGAAATTGGACAGC
DR	TGGTACCCGGGAGCTCGAACATCATCTTATATAACCGCGTCTTTTGACAC
P-AF	CATCATCTTATATAACCGCGTCTTT
P-AR	TCCGATTTCTACTCCGTAGACGTCA
P-BF	GGTGATTTTGGTTCCACAGTTGTTT
P-BR	AGGTTTTTGATGGCGAAGAATTTTT
P-CF	TTACAAGTTTAGCATCTCCGGCTTC
P-CR	TTCATAGTATCCATTGGCCGAGTAC
P-DF	TACCTCCCTCTACTTGAAATTGGAC
P-DR	CATCATCTTATATAACCGCGTCTTT
fiber 1-F	ATGTCGGCCCTAATCGCCTC
fiber 1-R	TTAGGGGCCCGGAGCATTG
penton-F	ATGTGGGGGTTGCAGCCGCCGACG
penton-R	CTACTGCAAGGTCGCGGAACTCAG

^*a*^ Underlining indicates homology arms between viral and vector sequences.

### Construction of recombinant FAdV-4 expressing the EGFP gene (rFAdV-4-EGFP)

**Fig 2:**
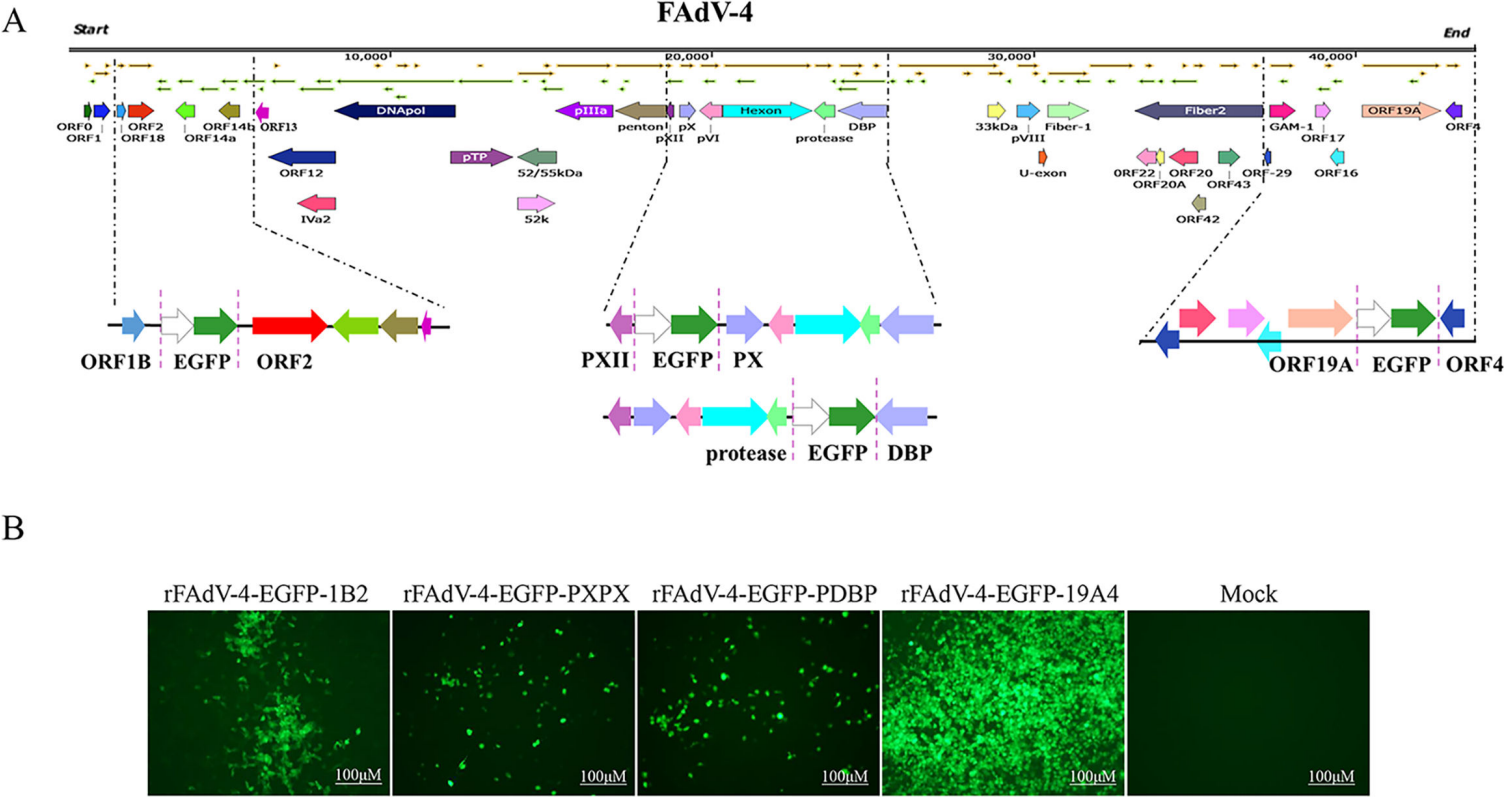
Construction and identification of four recombinant fowl adenovirus 4 expressing the EGFP gene (rFAdV-4-EGFP). (**A**) Construction of rFAdV-4-EGFP at four different positions between ORF1B and ORF2, PXII and PX, protease and DBP, and ORF19A and ORF4 positions. (**B**) Observation of EGFP expression in four rFAdV-4 via fluorescence microscopy (scale bar, 100 μm).

To identify the suitable insertion site for foreign gene expression, the EGFP expression cassette from the pEGFP-N1 plasmid was inserted at different positions in the FAdV-4 genome, including between ORF1B and ORF2 (Fragment A), PXII and PX (Fragment B), protease and DBP (Fragment C), and ORF19A and ORF4 (Fragment D) (Fig. 2A). The primer sequences used for construction are listed in Table 2. To rescue the recombinant viruses, the fragment containing EGFP was co-transfected with other fragment into LMH cells. Subsequently, the LMH cells were freeze-thawed twice and inoculated into LMH cells to generate rescued virus. The rescued viruses were named rFAdV-4-EGFP-1B2, rFAdV-4-EGFP-PXPX, rFAdV-4-EGFP-PDBP, and rFAdV-4-EGFP-19A4.

**TABLE 2:** Primers for the construction of recombinant FAdV-4-EGFP^*a*^

Name	Primer sequence (5′−3′)
ORF1B/ORF2-F	ATTGTAACTGATTGTAACTGCGTTACATAACTTACGGTAAATGG
ORF1B/ORF2-R	CACATTTAATACATTTCAATTAAGATACATTGATGAGTTTGGACA
pOK12-A-1b2-FP	ATTGAAATGTATTAAATGTGCTCGCATTA
pOK12-A-1b2-RP	CAGTTACAATCAGTTACAATCAGTTACAA
pXII/pX-F	GTTCGAACGACTTGTTATAGCCGTTACATAACTTACGGTAAATGG
pXII/pX-R	TCGTTACCAAAGTCACGAAGTAAGATACATTGATGAGTTTGGACA
pOK12-B-PXPX-FP	CTTCGTGACTTTGGTAACGACTTTGTAC
pOK12-B-PX-PX-RP	GCTATAACAAGTCGTTCGAACTAGAATCG
protease/DBP-F	AGCATAGCGACTCCTTTATTCGTTACATAACTTACGGTAAATGG
protease/DBP-R	GTTTATTGCCTTCCTTTCACTAAGATACATTGATGAGTTTGGACA
pOK12-C-PDBP-FP	GTGAAAGGAAGGCAATAAACATTCGTCA
pOK12-C-PDBP-RP	GTACAACAAGCATAGCGACTCCTTTATT
19A/4-F	TAAGAGCATGAATCAACTCGCGTTACATAACTTACGGTAAATGG
19A/4-R	GAAAGGAATGGGCGGACACTAAGATACATTGATGAGTTTGGACA
pOK12-D-19A4-FP	GTGTCCGCCCATTCCTTTCTCTATATATT
pOK12-D-19A4-RP	CGAGTTGATTCATGCTCTTATCTTTATTG

^*a*^ Underlining indicates homology arms between viral and vector sequences.

### Construction of rFAdV-4-HiBiT

The HiBiT gene was cloned into the pEGFP-N1 plasmid, and the EGFP ORF was replaced to form the HiBiT-expressing cassette. The HiBiT-expressing cassette is composed of an upstream cytomegalovirus promoter, the HiBiT sequence, a downstream poly (A) and part of the pEGFP-N1 vector sequence. The HiBiT cassette was inserted into the ORF19A and ORF4 to generate pOK12-D-HiBiT. LMH cells were co-transfected with 1 μg each of A, B, C, and D-HiBiT fragments. The rescued virus was named rFAdV-4-HiBiT (Fig. 4A). The primers used for cloning are listed in Table 3.

**TABLE 3:** Primers for constructing and detection of the genetic stability of rFAdV-4-HiBiT^*a*^

Name	Primer sequence (5′−3′)
19A/4-F	TAAGAGCATGAATCAACTCGCGTTACATAACTTACGGTAAATGG
19A/4-R	GAAAGGAATGGGCGGACACATGGGGCGGAGAATGGGCGGAA
pOK12-D-19A4-FP	GTGTCCGCCCATTCCTTTCTCTATATATT
pOK12-D-19A4-RP	CGAGTTGATTCATGCTCTTATCTTTATTG
FAdV-4 HiBiT-F	ATTGCCCTTAGAAGGAGAGGG
FAdV-4 HiBiT-R	TACCTATCTATTTCTTCTAGGGT

^*a*^ Underlining indicates homology arms between viral and vector sequences.

### Indirect immunofluorescent antibody (IFA) analysis

LMH cells were infected with the viruses at an MOI of 0.1. The cells were fixed with 4% paraformaldehyde for 15 min and then permeabilized with 0.1% Triton X-100 for 15 min. The cells were incubated with anti-FAdV-4 fiber 1 polyclonal antibody (1:200) for 1.5 h at 37°C. Then, the cells were washed three times with PBS and stained with anti-rabbit IgG FITC (1:300) at 37°C for 1 h. Finally, the cells were analyzed under a fluorescence microscope to observe green fluorescence.

### Western blotting analysis

LMH cells were lysed for 15 min using ice-cold RIPA (Solarbio) at 4°C. Proteins were loaded onto a 10% SDS-PAGE gel and transferred to a polyvinylidene fluoride (PVDF) membrane. The PVDF membrane was blocked with 5% milk in TBST for 1 h and incubated with the rabbit anti-FAdV-4 fiber 1, mouse anti-EGFP, mouse anti-HA and mouse anti-β-actin antibodies. Then, the membrane was incubated with anti-rabbit IgG and/or anti-mouse IgG secondary antibody. The protein bands were visualized using an Azure c600 imager.

### Virus growth kinetics

LMH cells were infected with recombinant virus at an MOI of 0.1 and incubated for 1 h at 37°C with 5% CO_2_. The cells were washed three times with PBS, and then supplemented with maintenance medium containing 2% FBS. The cells were harvested every 12 hpi (24, 48, 72, 96, and 120 h). The viral titers were determined using TCID_50_ titration. The mean values and standard deviations (SDs) were calculated from the results of three independent experiments.

### Flow cytometry

LMH cells were infected with various rFAdV-4-EGFP at an MOI of 0.1. Cells were harvested and washed with PBS at 24, 48, and 72 hpi. The cells were resuspended in 1 mL PBS and transferred to 5 mL fluorescence-activated cell sorting (FACS) tubes. The mean fluorescence intensity of EGFP-positive cells (10,000 events captured per sample) was assessed via FACS analysis (34).

### HiBiT stability during passaging

rFAdV-4-HiBiT was serially passaged into LMH cells 10 times. Viral DNA was extracted from the cell culture supernatants from 6th through 10th passages for PCR analysis using the FAdV-4 HiBiT-F/R primer pair (Table 3) to evaluate the stability of the foreign sequence. The RCR reaction underwent 98°C for 3 min; 98°C for 10 s, 55°C for 30 s, 72°C for 2 min, 32 cycles; and 72°C for 10 min. The amplified products were detected using agarose gel electrophoresis. The amplified product from the 10th LMH passage was subjected to Sanger sequencing.

### Fluorescence activity assay

LMH cells were infected with rFAdV-4-HiBiT at an MOI of 0.1. The HiBiT activity in relative luminescence unit (RLU) was measured using the Nano-Glo HiBiT Extracellular Detection Reagent (Promega) according to the manufacturer’s instructions.

### rFAdV-4-HiBiT neutralization assay

Forty-four positive serum samples were collected from SPF chickens infected with FAdV-4, whereas six FAdV-4-negative chicken serum samples were collected prior to infection. After sera were heat-inactivated at 56°C for 30 min, each serum sample was diluted twofold in series in DMEM from 1:2 through 1:512. Each diluted serum was mixed with an equal volume of 200 TCID_50_ rFAdV-4-HiBiT and incubated at 37°C for 1 h. The virus-antibody mixtures were transferred to 96-well plates seeded with monolayered LMH cells and set up negative control (normal cells) and positive control (200 TCID_50_ virus solution). The luciferase activity was measured after incubation for 24 h at 37°C, four replicate wells per dilution. The relative luciferase signal was calculated by normalizing the luciferase signals of serum-treated groups to those of the no-serum controls. The concentration that reduced the luciferase signal by 50% was estimated using a four-parameter logistic regression model with the Prism 8 software (35). All the serum samples were tested in parallel using the traditional CPE neutralizing antibody method (36).

### Applicability of rFAdV-4-HiBiT to screen antiviral protein

LMH cells were transfected with negative control plasmids (pCAGGS-HA) or expressing plasmids pCAGGS-ANXA5-HA, pCAGGS-SQSTM1-HA, pCAGGS-HSP90AA1-HA, pCAGGS-CCT5-HA, and pCAGGS-CD74-HA. After transfection for 24 h, the cells were infected with rFAdV-4-HiBiT at 0.1 MOI for 48 h and assayed for HiBiT activity. As a positive control, LMH cells were pretreated with 100 ng of chIFN-α for 24 h and infected with rFAdV-4-HiBiT. HiBiT activity (rFAdV-4-HiBiT) was determined as described previously.

### Applicability of rFAdV-4-HiBiT to screen antiviral drug

The cytotoxicity of Ribavirin, Apigenin, Honeysuckle flower, and *L. lucidum* was assessed using an MTT Reagent Kit (37). Briefly, LMH cells were treated with each reagent at a range of concentrations and incubated for 48 h at 37°C. Dimethyl sulfoxide was used as the negative control. Cell viability was tested using an MTT Reagent Kit. The 50% cytotoxic concentration (CC_50_) was calculated using the GraphPad Prism (version 8.0) software.

Based on the results of the cytotoxicity assay, the cells were infected with rFAdV-4-HiBiT and were subsequently treated with various concentrations of drugs. After 48 hpi, HiBiT activity (rFAdV-4-HiBiT) was determined as described above. Anti-rFAdV-4-HiBiT activity of each drug was expressed as the median inhibition concentration (IC_50_) calculated by fitting dose-response curves using GraphPad Prism (version 8.0) software to determine the drug concentration required to achieve a 50% RLU reduction (27, 35, 38).

### Statistical analysis

The data were analyzed using the GraphPad Prism (version 8.0) software and were presented as means ± SD. Statistical significance between different groups was determined using one-way ANOVA. Differences were considered not significant (ns) *P* > 0.05, *P < 0.05, **P < 0.01, and ***P < 0.001.

## RESULTS

### Development of reverse genetics system for FAdV-4

To establish a reverse genetics system for the FAdV-4 Y17215-1 strain, the PCR products of four fragments (Fig. 1B) were gel-purified and co-transfected (~1 ug each) into LMH cells. The rescued virus rFAdV-4 exhibited typical CPE similar to those of the parental virus, including cell rounding, wrinkling, and detachment, whereas no CPE was observed in the negative control group (Fig. 1C). IFA was employed to detect the expression of FAdV-4 fiber 1 protein, which exhibited specific green fluorescence in both rFAdV-4 and FAdV-4-infected LMH cells (Fig. 1D). The PCR results showed that the expected bands for fiber 1 or penton genes were successfully amplified from LMH cells infected with rFAdV-4 and FAdV-4 (Fig. 1E). Western blotting results also confirmed that the fiber 1 protein was expressed (52 kDa) (Fig. 1F) and demonstrated that rFAdV-4 was successfully rescued. The growth kinetic results showed that the rFAdV-4 exhibits similar growth kinetics to its parental virus (FAdV-4), with both reaching peak replication at 72 h (Fig. 1G). These results clearly indicated that a reverse genetics system for FAdV-4 has been successfully established.

### Screening the optimal insertion site for expressing foreign genes

**Fig 3:**
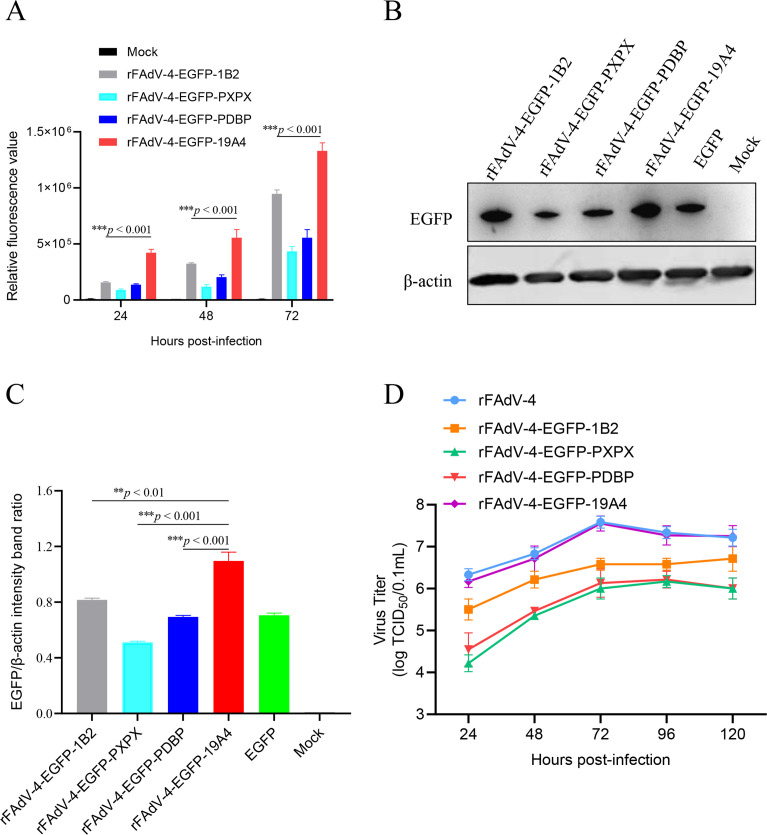
Biological characterization of four recombinant FAdV-4 expressing EGFP gene (rFAdV-4-EGFP). (**A**) Relative fluorescence values of LMH cells infected with rFAdV-4-EGFP-1B2, rFAdV-4-EGFP-PXPX, rFAdV-4-EGFP-PDBP, and rFAdV-4-EGFP-19A4 strains was analyzed by FACS. (**B**) The expression of EGFP in four rFAdV-4 was analyzed by western blotting. (**C**) The intensity band ratio of EGFP/β-actin normalized to the control. (**D**) Replication kinetics of four recombinant fluorescent viruses (**P < 0.01 and ***P < 0.001).

To screen for the optimal insertion site within FAdV-4 genome, the EGFP gene was inserted at four different sites between ORF1B and ORF2, PXII and PX, protease and DBP, and ORF19A and ORF4 genes, respectively (Fig. 2A). Four recombinant viruses were generated using the reverse genetics system, designated as rFAdV-4-EGFP-1B2, rFAdV-4-EGFP-PXPX, rFAdV-4-EGFP-PDBP, and rFAdV-4-EGFP-19A4. LMH cells were infected with each of the four recombinant viruses expressed green fluorescence, while uninfected LMH cells did not express green fluorescence (Fig. 2B), indicating that the four recombinant viruses were able to successfully express EGFP. In the FACS, compared with LMH cells infected with rFAdV-4-EGFP-1B2, rFAdV-4-EGFP-PXPX, or rFAdV-4-EGFP-PDBP, LMH cells infected with rFAdV-4-EGFP-19A4 exhibited significantly higher fluorescence intensity (approximately 2.68- to 4.69-fold) at 24, 48, and 72 hpi, respectively. (Fig. 3A). Western blotting analysis showed that the EGFP expression levels of rFAdV-4-EGFP-19A4 were 1.34-, 2.14-, and 1.58-fold higher than those of rFAdV-4-EGFP-1B2, rFAdV-4-EGFP-PXPX, and rFAdV-4-EGFP-PDBP, respectively (Fig. 3B and C). Furthermore, the growth kinetics results showed that rFAdV-4-EGFP-19A4 had similar replication kinetics to its parental virus in LMH cells, with a viral titer of approximately 10^7.5^ TCID_50_/0.1 mL. In contrast to rFAdV-4-EGFP-19A4, the viral titers of rFAdV-4-EGFP-1B2, rFAdV-4-EGFP-PXPX, and rFAdV-4-EGFP-PDBP were 10-, 89-, and 39-fold reduced, respectively (Fig. 3D). The findings suggest that the region between the ORF19A and ORF4 genes is an optimal site for the expression of foreign genes.

### Generation of rFAdV-4-HiBiT

**Fig 4:**
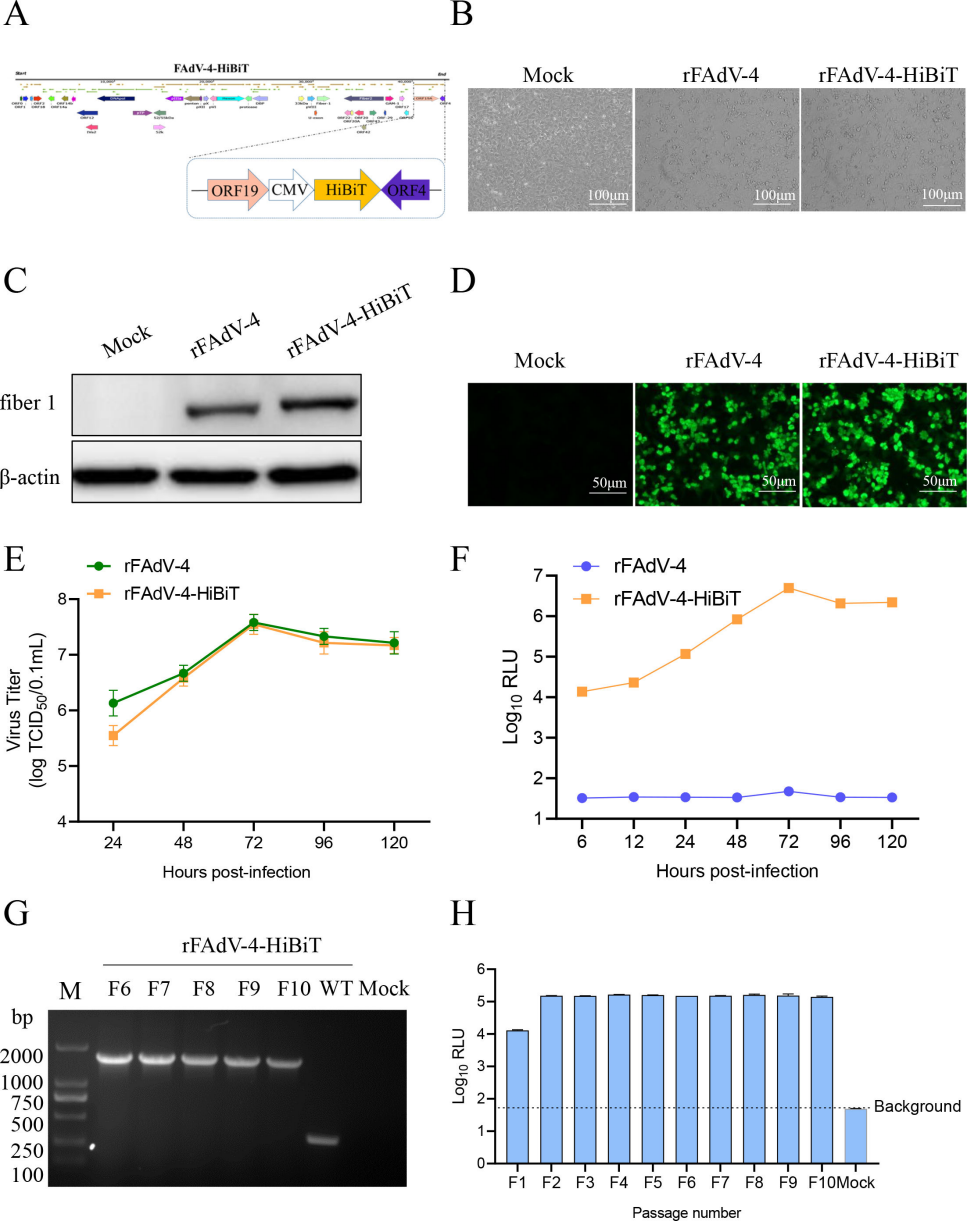
Characterization and genetic-stability analysis of recombinant FAdV-4 expressing HiBiT gene (rFAdV-4-HiBiT). (**A**) Schematics for constructing the rFAdV-4-HiBiT strain. (**B**) Cytopathic eﬀect (CPE) of the LMH cells infected with the rFAdV-4 and rFAdV-4-HiBiT viruses after 24 hpi. (**C**) Positive western blotting bands of fiber 1 protein of rFAdV-4 and rFAdV-4-HiBiT. (**D**) Immunofluorescence analysis of the rFAdV-4 and rFAdV-4-HiBiT strains (scale bar, 50 μm). (**E**) Growth kinetics of rFAdV-4 and rFAdV-4-HiBiT. (**F**) Time-course results of the HiBiT gene expression. (**G**) The HiBiT gene was amplified by PCR using total DNAs from rFAdV-4-HiBiT-infected cells as the template. (**H**) The results of luciferase activity detection in different generations of rFAdV-4-HiBiT strains.

To obtain a quantifiable recombinant reporter FAdV-4, LMH cells were co-transfected with fragments A, B, C, and D-HiBiT. After the second passage, LMH cells exhibited characteristic lesions (Fig. 4B). Western blotting confirmed the expression of the fiber 1 protein (52 kDa) in LMH cells infected with rFAdV-4-HiBiT (Fig. 4C). The IFA results further showed green fluorescent signals (Fig. 4D), indicating that rFAdV-4-HiBiT was successfully rescued.

### Replication properties and genetic stability of the rFAdV-4-HiBiT

To assess the replication properties of rFAdV-4-HiBiT, the growth kinetics of the virus were measured. The results showed that rFAdV-4-HiBiT and rFAdV-4 exhibited similar growth kinetics in LMH cells (Fig. 4E). The results of the HiBiT activity assay showed that rFAdV-4-HiBiT reached its maximum values (Log_10_RLU = 6.68) at 72 hpi (Fig. 4F). To further evaluate the genetic stability of the rFAdV-4-HiBiT, 10 serial passages were performed after inoculation into LMH cells (MOI = 0.1). The PCR results showed that the expected sequence sizes (1,961 bp) were successfully amplified from the 6th to 10th passages of rFAdV-4-HiBiT (Fig. 4G). In addition, the genetic stability results demonstrated that the luciferase activity of rFAdV-4-HiBiT remained consistent during passaging (Fig. 4H), suggesting that the rFAdV-4-HiBiT has high genetic stability.

### Establishment of a luciferase-based rFAdV-4-HiBiT neutralization assay

**Fig 5:**
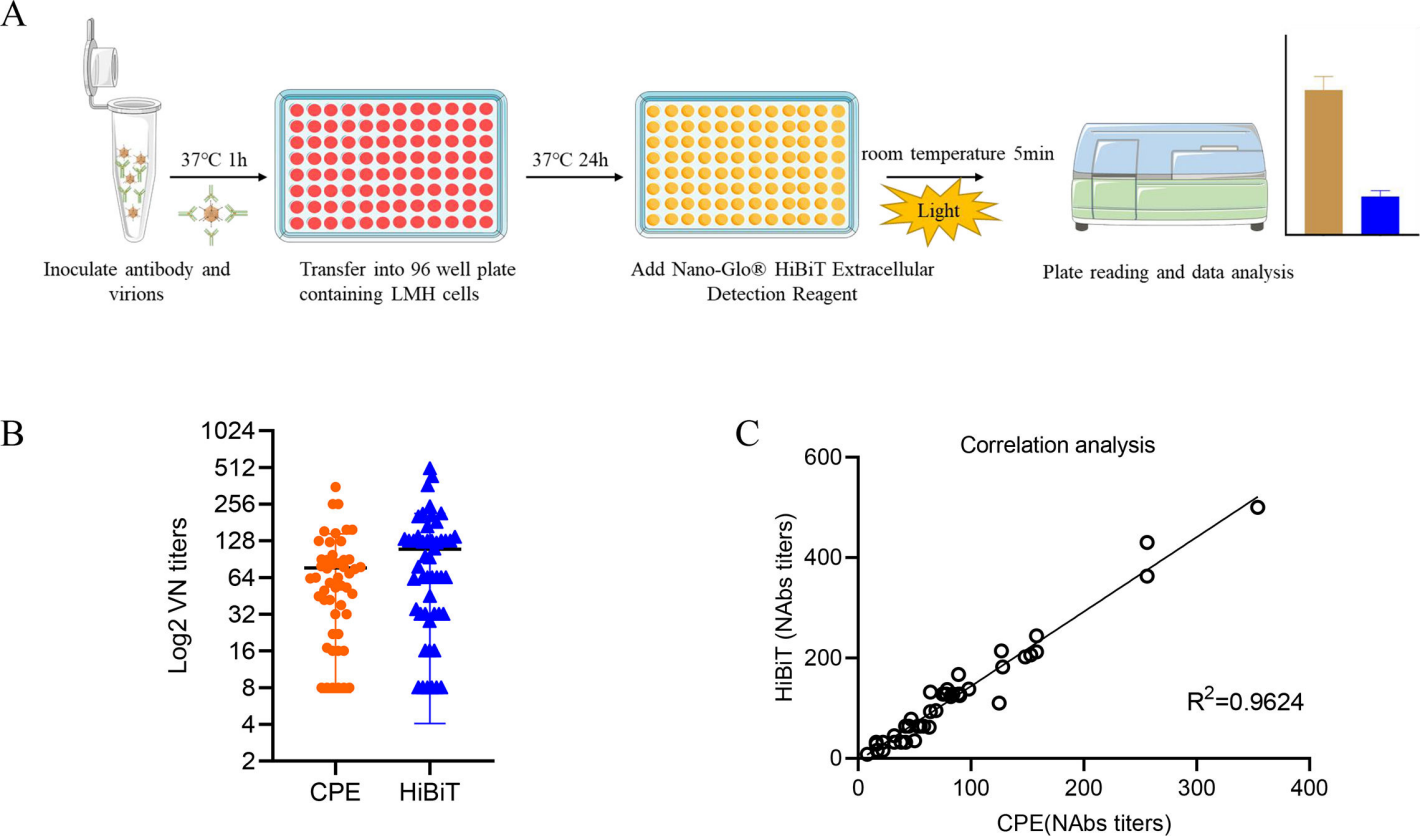
Application of rFAdV-4-HiBiT for neutralizing assay. (**A**) Schematic diagram of luciferase-based determination of neutralizing antibody. (**B**) Summary of neutralizing titers as measured by conventional neutralization (cytopathic eﬀect [CPE]) and rFAdV-4-HiBiT neutralization (HiBiT) assay. (**C**) Correlation between the luciferase-based neutralizing antibody test and the traditional CPE neutralizing antibody test.

The rFAdV-4-HiBiT was used to measure anti-FAdV-4 neutralizing antibody titer in chicken serum samples (Fig. 5A). All FAdV-4 positive serum (samples #1–44) showed positive HiBiT of 16–500 (NAbs titers) and CPE of 16–354 (NAbs titers), while all FAdV-4-negative serum (samples #45–50) showed negative HiBiT (NAbs titers) ≤8 and CPE (NAbs titers) ≤8 (Fig. 5B). The coefficients of determination (*R*^2^) between the results of the luciferase activity and the conventional neutralization assay were 0.9624 (Fig. 5C), which demonstrated the accuracy of the luciferase-based neutralizing antibody potency assay.

### Application of rFAdV-4-HiBiT for screening antiviral proteins

**Fig 6:**
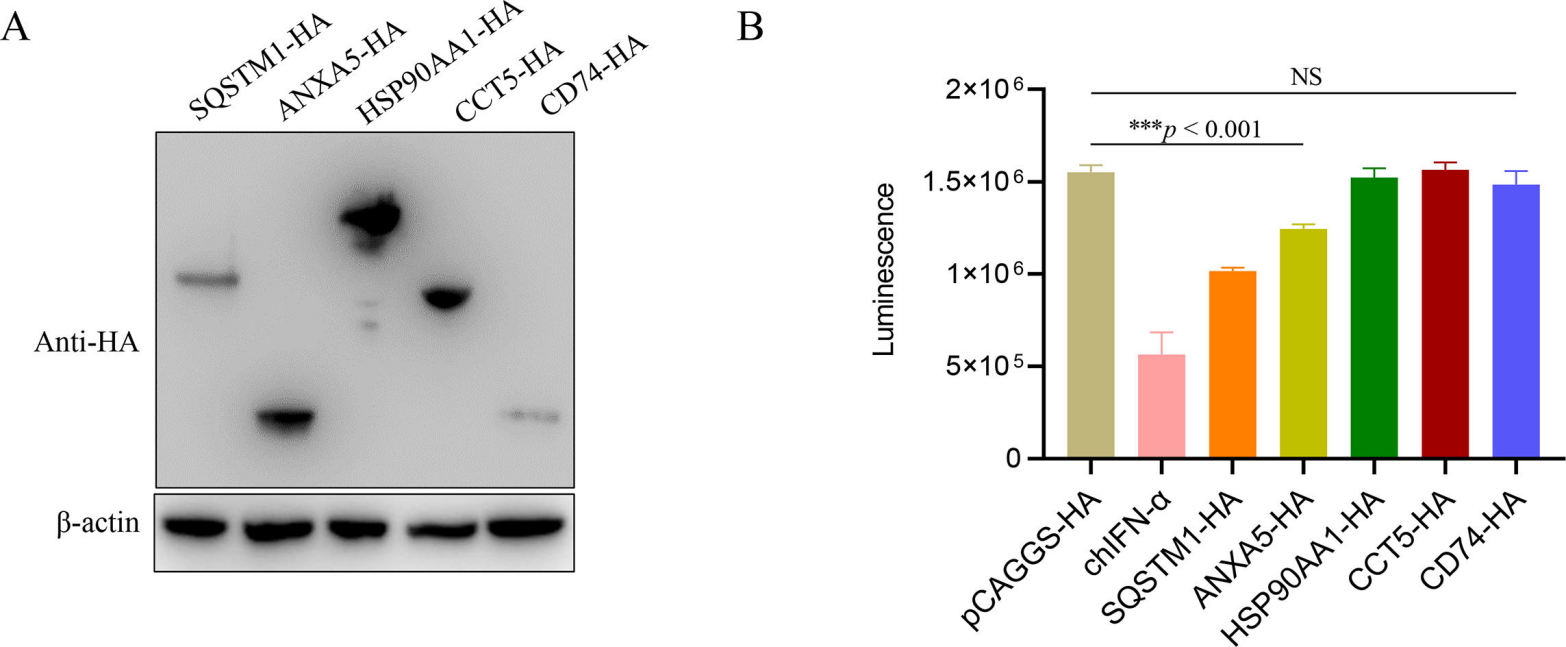
Screening of antiviral protein using rFAdV-4-HiBiT. (**A**) Antiviral proteins were detected using western blotting. (**B**) Screening of antiviral proteins against rFAdV-4-HiBiT based on luciferase activity. Statistical differences were determined using one-way ANOVA method. Error bars represent standard deviations. Each sample was run in triplicate (NS = not significant and ***P < 0.001).

Western blotting assay was performed to investigate the expression of ANXA5, SQSTM1, HSP90AA1, CCT5, and CD74 in LMH cells. The results showed that ANXA5 (35 kDa), SQSTM1 (62 kDa), HSP90AA1 (80 kDa), CCT5 (59 kDa), and CD74 (34 kDa) were expressed at the expected sizes (Fig. 6A). Subsequently, each of the five recombinant plasmids described above was transfected into LMH cells, and a negative control group (pCAGGS-HA) and a positive control group (chIFN-α protein) were set up. After 24 h of transfection, the cells were infected with rFAdV-4-HiBiT (0.1 MOI). Notably, the overexpression of SQSTM1 and ANXA5 significantly decreased HiBiT activity in cells infected with rFAdV-4-HiBiT, whereas overexpressing HSP90AA1, CCT5, and CD74 did not show significant differences in their corresponding luciferase activity compared to the pCAGGS-HA group (Fig. 6B). These results suggest the feasibility of using rFAdV-4-HiBiT to screen for antiviral proteins.

### Applicability of rFAdV-4-HiBiT for antiviral drug screening

**Fig 7:**
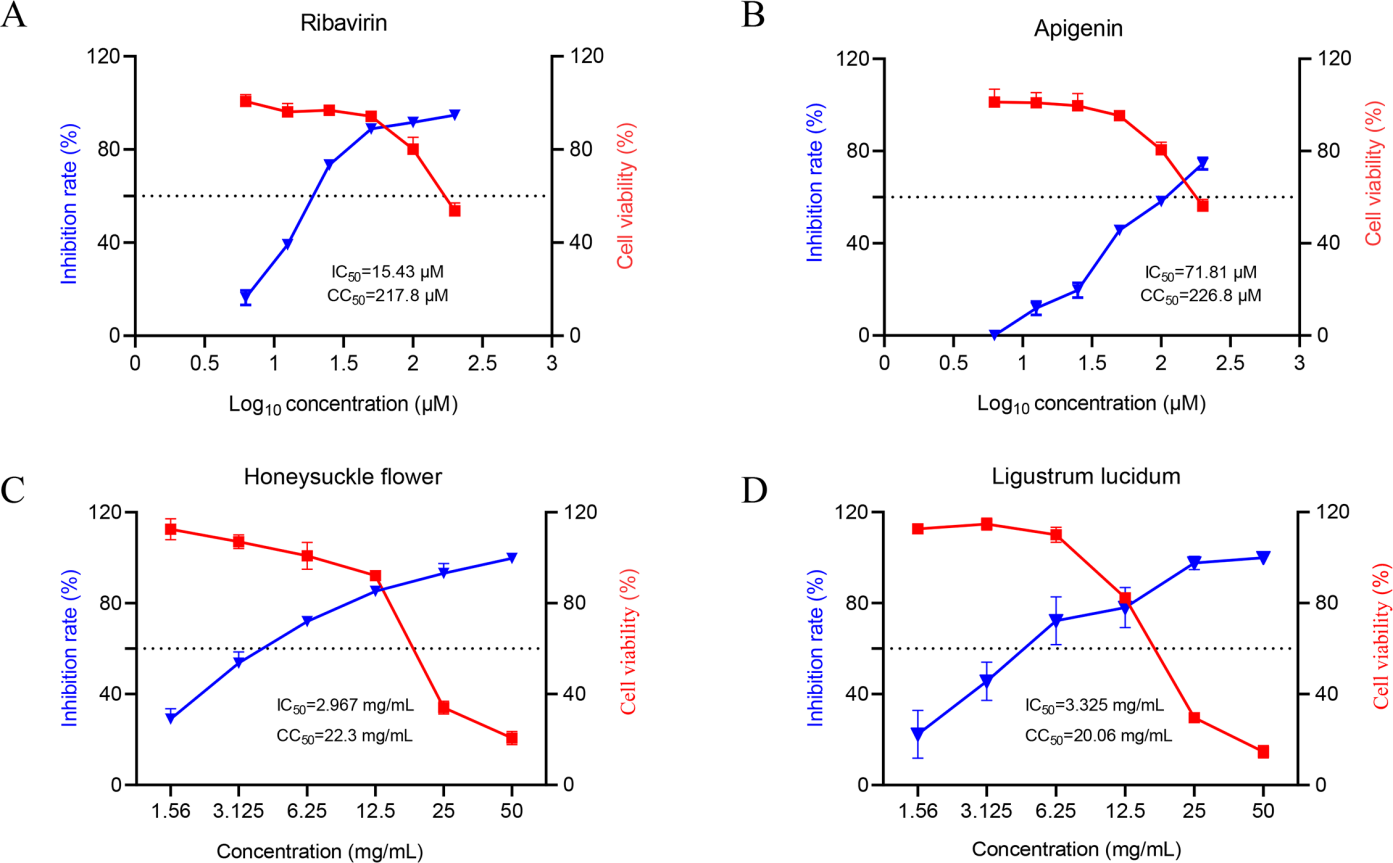
Antiviral testing of Ribavirin, Apigenin, Honeysuckle flower, and *Ligustrum lucidum* based on rFAdV-4-HiBiT. (**A–D**) LMH cells infected with the rFAdV-4-HiBiT at MOI 0.1 and incubated at 37°C for 1 h. Supernatants were replaced with DMEM containing different concentrations of drugs. At 48 hpi, the inhibitory effect of the drug on the virus was analyzed by HiBiT activity detection, and cell activity was analyzed by MTT assay. The figure displays dose-response curves for infectivity (blue) and cellular activity (red). The IC_50_ and CC_50_ values for each compound were calculated using a four-parameter logistic nonlinear regression in GraphPad Prism (version 8.0) software. (**A**) Ribavirin. (**B**) Apigenin. (**C**) Honeysuckle flower. (**D**) *L. lucidum*. Data were expressed as means ± SD (*n* = 3).

To verify whether rFAdV-4-HiBiT could be applied for antiviral drug screening, four drugs (Ribavirin, Apigenin, Honeysuckle flower, and *L. lucidum*) were tested for their antiviral activity. LMH cells were inoculated with rFAdV-4-HiBiT and treated with various concentrations of Ribavirin, Apigenin, Honeysuckle flower, and *L. lucidum*. At 48 hpi, the luciferase activities of rFAdV-4-HiBiT infected-LMH cells supernatants were measured. The CC_50_ values of Ribavirin, Apigenin, Honeysuckle flower, and *L. lucidum* were 217.8 μM, 226.8 μM, 22.3 mg/mL, and 20.06 mg/mL, while the IC_50_ values were 15.43 μM, 71.81 μM, 2.967 mg/mL, and 3.325 mg/mL, respectively (Fig. 7A through D). Correspondingly, the selectivity index (SI) values of Ribavirin, Apigenin, Honeysuckle flower, and *L. lucidum* are 14.1, 3.15, 7.5, and 6.0, respectively (SI value ≥ 4 is considered antiviral) (39), which demonstrated that Ribavirin, Honeysuckle flower, and *L. lucidum* effectively inhibited the replication of FAdV-4 *in vitro*.

## DISCUSSION

Reverse genetics system is a powerful tool for engineering the DNA or RNA virus genome. Due to the large FAdV-4 genome, CRISPR/Cas9 gene editing and CopyControl fosmid library production technologies are currently employed to achieve viral modification. Guo et al. co-transfected LMH cells with sgRNA targeting the FAdV-4-EGFP fiber 2 gene and a donor plasmid. Then, the recombinant virus FAdV-4-HA expressing the HA gene of H9N2 influenza virus was prepared by continuous blind passage, limited dilution, and plaque purification (40). In addition, Zhang et al. constructed a fosmid library plasmid of the FAdV-4 HLJFAd15 and rescued the rFAdV-4 HLJFAd15 using a two-step recombination method mediated by the rpsL-neo antibiotic selection cassette (11). However, generating recombinant FAdV in these studies required additional purification and multiple homologous recombination steps, which increased the risk of mutations. To simply and rapidly generate the full-length genome of FAdV-4, four plasmids covering the full-length genome of FAdV-4 were generated in this study. In the process of foreign gene insertions, deletions, or mutations, only a single plasmid requires modification to obtain the complete full-length sequence. Furthermore, during virus rescue using CRISPR/Cas9 technology, inoculated parental viruses may interfere with the efficient propagation of the generated recombinant viruses, which may increase the risk of hybridization of rescued recombinant viruses. Conversely, the rescue virus was obtained in this study by co-transfection of only four fragments into LMH cells without removing the parental virus, which significantly enhanced the efficiency of viral rescue.

Previous studies have shown that adenovirus genomes are sufficiently large to accommodate the stable expression of foreign genes. Pei et al. successfully rescued two recombinant fluorescent stable viral strains by replacing the EGFP gene with ORF16 and ORF17 of the FAdV-4 ON1 strain, respectively, whose titers were 10^2^- to 10^3^-fold lower than those of the parental virus (41). Furthermore, the substitution of EGFP in the L1 (ORF0, ORF1, ORF1b, and ORF2) and L2 (ORF24, ORF14a, and ORF14) domains resulted in an approximately 10-fold decrease in viral titers (42). These results indicate that inappropriate substitution sites can reduce viral replication, although FAdV-4 can still be used as a viral vector for foreign gene expression. Previous studies have demonstrated that the 1,966 bp deletion site is a natural site suitable for inserting foreign sequences (43). However, this insertion site alone is not suitable for the development of multivalent vaccines for poultry. Considering the extensive genome of FAdV-4, we analyzed the impact of inserting the EGFP gene at different sites on viral replication. It was found that when the EGFP gene was inserted between the ORF19A and ORF4 genes of the FAdV-4, it did not affect the level of viral replication. Furthermore, compared to other insertion sites, this site exhibited the highest EGFP expression levels. Critically, insertion of a foreign gene between the ORF19A and ORF4 genes did not delete the viral-encoded proteins or damage the particle integrity of the virus. To the best of our knowledge, this is the first study to screen for optimal insertion sites in the spacer region of FAdV-4. It improves our knowledge of the FAdV-4 genome and provides novel targets for developing multivalent vector vaccines for poultry.

Conventional FAdV-4 neutralization assays are time-consuming and require a combination of lesion observations to evaluate neutralizing antibody titers, which are often subjective. Although rFAdV-4-EGFP allows for real-time visualization, it often suffers from background interference caused by cellular autofluorescence (32). In contrast, HiBiT tag offers lower background noise; furthermore, neutralizing antibody titers determined by the rFAdV-4-HiBiT assay exhibit a high relevance to those measured by the conventional CPE neutralization assay. However, the testing period was reduced from 120 to 24 h, which significantly simplified the detection procedure. Additionally, due to the amplifying nature of the HiBiT enzyme, the rFAdV-4-HiBiT assay exhibits greater sensitivity than those of fluorescent EGFP virus assays (44, 45). These findings further indicate that the rFAdV-4-HiBiT offers great potential for rapid detection of neutralizing antibody titers.

Since there are no specific drugs available for the prevention and control of HHS, it is essential to screen antiviral drugs and proteins against FAdV-4. To evaluate the viability of rFAdV-4-HiBiT for antiviral screening, a proof-of-concept study was performed using four potential antiviral reagents: Ribavirin, Apigenin, Honeysuckle flower, and *L. lucidum*. Luciferase activities after treatment with these drugs were assayed after 48 hpi, and it was demonstrated that Ribavirin, Honeysuckle flower, and *L. lucidum* effectively inhibited the replication of FAdV-4 *in vitro*. Additionally, two antiviral proteins (SQSTM1 and ANXA5) that decreased FAdV-4 replication were successfully identified using rFAdV-4-HiBiT, demonstrating its suitability for screening antiviral agents. To the best of our knowledge, this is the first study on antiviral drug and protein screening using the HiBiT rFAdV-4 reporter virus, which is an effective and convenient tool for screening antiviral reagents, as it does not require CPE observation and plaque assays.

In summary, we successfully established an improved reverse genetics system for FAdV-4. Based on this system, we further developed a reporter rFAdV-4-HiBiT, which was used for the rapid detection of neutralizing antibodies and screening of antiviral compounds. Thus, this study provides valuable tools for antiviral agents development and vaccine evaluation.

## SUPPLEMENTAL MATERIAL

Supplemental material spectrum.03347-25-s0001.docx Supplementary HiBiT-expressing cassette sequence and rFAdV-4-HiBiT sequence.

ASM does not own the copyrights to Supplemental Material that may be linked to, or accessed through, an article. The authors have granted ASM a non-exclusive, world-wide license to publish the Supplemental Material files. Please contact the corresponding author directly for reuse.
