# Supplementary material for: Development of recombinant fowl adenovirus serotype 4 harboring the HiBiT-Tag reporter and its utility in antiviral research
Source: Microbiol Spectr. 2026 Jan 28;14(3):e03347-25. doi: 10.1128/spectrum.03347-25 (PMC12955450; doi:10.1128/spectrum.03347-25)
Supplement: Supplemental material — Supplementary HiBiT-expressing cassette sequence and rFAdV-4-HiBiT sequence. [file spectrum.03347-25-s0001.docx]

**Supplementary** **HiBiT-expressing cassette sequence and rFAdV-4-HiBiT sequence**

**HiBiT-expressing cassette sequence**

CGTTACATAACTTACGGTAAATGGCCCGCCTGGCTGACCGCCCAACGACCCCCGCCCATTGACGTCAATAATGACGTATGTTCCCATAGTAACGCCAATAGGGACTTTCCATTGACGTCAATGGGTGGAGTATTTACGGTAAACTGCCCACTTGGCAGTACATCAAGTGTATCATATGCCAAGTACGCCCCCTATTGACGTCAATGACGGTAAATGGCCCGCCTGGCATTATGCCCAGTACATGACCTTATGGGACTTTCCTACTTGGCAGTACATCTACGTATTAGTCATCGCTATTACCATGGTGATGCGGTTTTGGCAGTACATCAATGGGCGTGGATAGCGGTTTGACTCACGGGGATTTCCAAGTCTCCACCCCATTGACGTCAATGGGAGTTTGTTTTGGCACCAAAATCAACGGGACTTTCCAAAATGTCGTAACAACTCCGCCCCATTGACGCAAATGGGCGGTAGGCGTGTACGGTGGGAGGTCTATATAAGCAGAGCTGGTTTAGTGAACCGTCAGATCCGCTAGCGCTACCGGACTCAGATCTCGAGCTCAAGCTTCGAATTCTGCAGTCGACGGTACCGCGGGCCCGGGATCCACCGGTCGCCACC**ATG****GTGAGCGGCTGGCGGCTGTTCAAGAAGATTAGCTTA**AGCGGCCGCGACTCTAGATCATAATCAGCCATACCACATTTGTAGAGGTTTTACTTGCTTTAAAAAACCTCCCACACCTCCCCCTGAACCTGAAACATAAAATGAATGCAATTGTTGTTGTTAACTTGTTTATTGCAGCTTATAATGGTTACAAATAAAGCAATAGCATCACAAATTTCACAAATAAAGCATTTTTTTCACTGCATTCTAGTTGTGGTTTGTCCAAACTCATCAATGTATCTTAAGGCGTAAATTGTAAGCGTTAATATTTTGTTAAAATTCGCGTTAAATTTTTGTTAAATCAGCTCATTTTTTAACCAATAGGCCGAAATCGGCAAAATCCCTTATAAATCAAAAGAATAGACCGAGATAGGGTTGAGTGTTGTTCCAGTTTGGAACAAGAGTCCACTATTAAAGAACGTGGACTCCAACGTCAAAGGGCGAAAAACCGTCTATCAGGGCGATGGCCCACTACGTGAACCATCACCCTAATCAAGTTTTTTGGGGTCGAGGTGCCGTAAAGCACTAAATCGGAACCCTAAAGGGAGCCCCCGATTTAGAGCTTGACGGGGAAAGCCGGCGAACGTGGCGAGAAAGGAAGGGAAGAAAGCGAAAGGAGCGGGCGCTAGGGCGCTGGCAAGTGTAGCGGTCACGCTGCGCGTAACCACCACACCCGCCGCGCTTAATGCGCCGCTACAGGGCGCGTCAGGTGGCACTTTTCGGGGAAATGTGCGCGGAACCCCTATTTGTTTATTTTTCTAAATACATTCAAATATGTATCCGCTCATGAGACAATAACCCTGATAAATGCTTCAATAATATTGAAAAAGGAAGAGTCCTGAGGCGGAAAGAACCAGCTGTGGAATGTGTGTCAGTTAGGGTGTGGAAAGTCCCCAGGCTCCCCAGCAGGCAGAAGTATGCAAAGCATGCATCTCAATTAGTCAGCAACCAGGTGTGGAAAGTCCCCAGGCTCCCCAGCAGGCAGAAGTATGCAAAGCATGCATCTCAATTAGTCAGCAACCATAGTCCCGCCCCTAACTCCGCCCATCCCGCCCCTAACTCCGCCCAGTTCCGCCCATTCTCCGCCCCAT

Underlining indicates HibiT squence

**rFAdV-4-HiBiT sequence**

CTCATCATCTTATATAACCGCGTCTTTTGACACACTTACAACCGCCGCGCGCAGCGCGGCTGAGTCATTGCATGAATTTCCGGGTTTGCACACTAGGGTTCTGAACTTTGATCTCATGACGCCAGTTTCGCTTTCGCGTGTAGTTCGAAACTGGCAACGCCTGGGCAAAGTCCTAACGTGATGTGTAATTTAAGTGTTTTTCCCGGTACATCCCATATCCTTGAAATGTTTACCGTTCGTTGCAAGTTTGAAGGTCATGTTTTATTCGCTTGTTTGTGTTAAATATTTACGTTTCAGTGTACTTCGGTCAGTAATTGTACTAATTGCGGGTCAAGGGACAGCCTTAATAGCACCACGAGTTTAGATATCGATCAGATACCATTACCCGTTGTCACGAAAGGTATTTTTCCACTCCGTTTAAGGGACGGTCCTCACGATATAAGTACTGGCGAGTCCGTCCTTTCGTTACAGATCTTCCTATGAGCTATAGGAGAACTGTCCCTCTTACTCGCTGTGCATTGCTTGACGCTGAAAACGCCGATATCGCGATCAGCGAGCCTTGCCATAACTTTGAAATTCAATTTCACCCTATTACGCCTCGGCGTGTATTCCTCCACTGCTTTGAACCCAACCGGTTTTGGACCGAAATCCTCTGGAACGGCACCGTGAAGCAGAGTGAACTGAACGCGGCGTTGGAGAAGATCGTTGAACTGTTATAGGTGAGGATTGTTTTCTGTACGTTTTTCGCGGTGGTGTCATCCCTTGACAATTTTCTCTCTTTTTCTACAAGGATGGCTGCCGTTTCTGCGTCTCCGTCTGTGCCTAAACTTCTATATAAGAAGGTTAAATCGGATGCTTTCGCTCCTGTCCGCATGTCGCCAGATGCAGCGGGTTTAGATTTGTTTAGTTGTGAGGACGTAGTGGTACCACCTCACGACAAGGCGCTGATTTCTACGGGGTTAATTTTAATCCTGCCTCCCAGCACTTATGGGAGAATCGCTCCTCGCTCGGGATTGGCTGCAAAGTTCTTTATAGATGTGGGGGCGGGTGTGATAGATGCTGATTATCGCGGTGAAGTGAAGGTGCTCTTGTTCAATTTTTCGCAACATGCGTTCAACGTTCGGAAGGGAGACCGTATCGCTCAGCTCGTTGTTGAGCGAATCTTTACACCGGAGCTCGAGGAGGTGTCCTCCGTTGATGACACGATCCGAGGAGGGAACGGATTCGGGTCGACTGGCACTGGTAGTGAAGCGATGTCCTCTCAAAGGACTCTACATTTGTGGCTTAAACCCAACGGTACTGGTTCTTCTTCGTAGGCTGATTCTGCGTGTCACGAACGCGCTTTCGACGAACCCGACTTTGCGGTGAAGATTTCCGCAGAAGTGGAGGCAGATAACGACGCTTACACGAATTGTTTTTGCGAGGGGGATACTGGAAGAAATTCTTGCTCTCTCTGTAGTTGTTCTCCGTGTGTAATTCTTTAGGTATGTTTCTTAAGATTCTAGTTCTCTTCTGTTTGATTGTTGTAGATTGTTACATCTTTCATACTCTCGGTGTTCACTGGCCCACCGCGATGCTTGTCGGCACTTGGATGCTTGTTGCCGAGCTGCTTAACGCCTGGGATGACTTCCGCGGTCGGCCTCGACTGCGATTTATGTCCATGCCGGACTTCGATGCCCTTTCACGCGCGGCTGATGAACTGGCTGCGATGCTAGATGAGAACCGTAACGCTGCTCCGGTTCAGCGCGGGGAAGAAGAAGTCTTTGAAGACAGCGACCGCGACCGCGATTCGGGAACCGATTGTAACTGATTGTAACTGATTGTAACTGATTGAAATGTATTAAATGTGCTCGCATTACAGATGGCTTCGGACCGTTACTGGGATTTAGTGAACTCGTTAATAAACAGGGGCATCGTGACGCGAGAACAATGGCAATCTGCGGATTTGGCTGAATATCGTCGGTACTCGAAGGGATACGTTAGGGGGTTCAGCGTACGCAAAGTTTTGCGCGATGTTATTAAACACATGTGCTGGACAAAAGTCCTCGGTGACTACTTAGTATGCCCTGTCGTGTGCCAAGACGACATTCATCTGAATCCGTTTTATGTAATTCTGATGAAAAACGGTTACAACCCCCGTGTCGTAGGAACAATTTTGCATAAGTGGTCCATGCTTACATCAAATAAGAATACTGTGTGGGTGTGGGGAGGTGCAGAAACGGGAGGGCCCTATTTGGCAGAGGCAATAGCTTATACCTCTCCTGTTGTCGGATGTGTGGATTGGCGGAATAGGGCCAATCCTTTTGCGCGCAATTATAATTGTTTGGTGTATTGGTTAGATGGTGGGCTTTTCCCTGAGAGCGCGATAGGGTTGTGTGAGCAAGTGTTGAGAGGGGAGGGAACTATGGTGGAGGTGGAGGAGGGTGCGACGGGAGAACGTAGATGGCGGGAGATTAACCGTACCCCCGTACTCATTTCTACGAGTCACGATGTAACGTTGACCTATGTGAAATATGGCCAAACTTGTAAGGATCATACTAATTCCCTGAGGAGTGCTATGTATGTGCTTCGTTTGACTGAACGCGTGGAACCCGGTTTTGTTATTACTTGTAATGACGCGCGTAAATTTGTAACCTGGGCTAGCAATAATCCACACATAAATGCAGAAGACATGTTGTAATTTATTCAAATTTGTAAAATGGAAAACATTCAATCTGAATAGTAAACTTCAATTGAGACAAGTTTCTCTGTATTTGTTCGCATGCGGCTTTTACTCGTGGGCTGAAATATTTGGGGAGGATCAGGATTCTGTAGCCGGTCGAGGTTAGGCCGATGCTTAAGTTTTCGAGAATGGATCTTTTGCAGAAGAGTAATTGTAAGCCCCAGAGCGGGTTGCAGTGACCCTCTTTGCGCAGACTGCAACGACACATGATGATGTCTTCGATGGGGCAGTGAGGGAAGGGATCGATGGGGATGTGTTCTTCGATAAATTCTTGAATGGTTTCGAACCAGTAGGCGAATACGAGTTGGCCTAGGCTCTCGCAGAACAGGGAATGGGGCATTCCGCAGGAACATTCGGATCGGATGGCGGGAGCACCGTGATCAGTCGTGTAGGGATAAAAGTTCATGAGGCGCAGTCTGACTTTGAAAGGGGGCATCTCGAGTTTGGGAAATAGTTCGTCTGCACTCACGTAGTCTCGACGGCAGAACGGGAACATGCGTTGACCCACGAGGAAGTTTAAGAAATGCGAGTCGAAGGCCAGATGGGAGGGGATTCGTTCGAAAAGGTGATTAAGTTGGATAGTCTTGGAGTGGAAAACAATTTTCTGTTAAAAGAAAATGAGGGTATAATTAATAAAGTAGATTTCCATGGCTCGTATTAAGCACCTATATGGAAAAGCGTCCTTCTTGATCCTCGGGATGCATATTTGCACCCCCTTTGGCGAAACTCGGAATGGGGTTTGTGATATTTCGAGGTAGGGTTCATTGTTGGGATTAACCGCAGTGATCAAGATGAATAGAAGTGTTTCGTCCTTGCACGCATGGCAGTGATCTAGGTAGCTATGGGCGAGAATGCCCAGATGTACCGGGAGTGGGATTTCGCGTGTAGTCGCTCTTATTTCGTCAACCCAACGTATATAGGTTTTCATTCGGAGGGAATGGCAGAAGAGACTATGAGGTTTGGGACAGGTACACCGGTATTTGAGCAACCATCTGGTTGCACAGTTTTTGGTTTCGTCGGCTTTGATATCGCGTAGACGTATTTCCACTCGGAATGATTTGAATCGAGGATAGAAAACGAATTCCTGAAAGGAATTGGGTGTTCCGGGAGTGAAAAACTGAGACTTTCCCACAAAGAAGGCGAAAAATTCTTTACTCGTGAGGACTTGGAATGACACAGAGTGAATAGGAGGCATGGAAATGATATGGGAGACGTGCCCGTTGCGGTCAGATCGTTTGTGAATGAATACAGGGTGTAGTTCTTCGGAGGGTATCTGCAACGAGAAGGATTACCTAGTGTGTATGTGCGTTGGGTAGGGGAAAGAATTGTGCATGTAATGCATGAAGCATGGAAGGTTAATGGCTTGGCTGGCGGATTCTGAAAGCCTGAGGATGATTTCACCGTCTTCGATGGAGAGTGGCGCTACACCATCGTTATGACAATGCTGTACTATTTCTGAGATTAGTTCGAGAGGCGTAGCTCCCGTGCAAAATGCGCAGTGGCCTAGTTCATCCGAGGTAAGGGGACCTAGATCTACGGGGAAGGGCTCTGCTCTGACTCCGTTTTGTAGAGCTTCTTTCCAAGAGTGGAAAAGGAGCTCTCTGAGAGACTGACAAAATAGGGAATTGCCGTCTCCGCAATTGCAACGCAGCTTTACTATCCATCCGAGTTCGGTATCCTCGGACGGTCTGAGAACACAGTAAACAGAGATGTCTGCTCTGAACGGTCTAACTCTAGGGTAAAGGCTCGTTTGTGAATACTCGTGGGGACCCGGGAAGGGGAATTGAATGTCACCGTTCATGAACTCTACGAATCGGTGGTCGAATAGCATTTGTATGTTTATGACATCGAAGAGGTCGTAGGTTCTGATGGTGAAACTGTGTTCAGAGTGTCGTACTTGATCGTCTTCGGGTGTCCAGCGAAAGATGGAAGTGGGCCGTCGTGTGGTCTGCAAGAGATTAGTAATAATAGACATGAATGCTGTGTGGCAGAATTTCGAGGGTTTTAAGGAAGCAGGACAGGTGGACGTAGTCGCGAAAAAATTTTGGGATGCCTAATCTAATGCGACCCTCTTGTCTCCTAAAGATGACGATACTTCGCCCTTGGAAGGTTTGTTTGACGATCTCGGTGAGAATTTCGTAGATCGTGTGGAAAGTGCAGTGGCTGCAGTGCCCTGTGCGCCTTTTAGCATAAGCGCGAATGGAGATGGGATGGCGTATGGGTACGGAGGCGAGGTGCTCCCGTACATCTAATCGCCATTGCTGGGCCACGTATTGATTAAGCGCTCGACAAAACAACGAGTATGGGTCTTTGCAACGGCAGTGGCTGTAGAGAATCCAGTAGGTGGATGATCTTTTTAGGTCCAGTTTGATGGTGATTTGGAAGGCAGGGAGGCATAGGTTGAGCTCTCGATGTGAGTAATGTTCTGCTGAGGGGAAGGGTAGGAGGGCTCGCCCGTTGATGAATTCGAGGAACCGTAGGTCGAAAAAGTGAATGGCGTCGCATATGTCTAGGGCGGGTCGACAGAGGATGGTGTAGTCGTGGCGGTGGTGAGGAGGGGAAGCCCTCGAACCGGTCAAAAGCACCATATCGGACACGGGGTTGATCTGAAACAAGATTTATTAGAGTGCATCAGAGGGCGAAGCCCGTATACAATTATCACAAATCTCATGGTCTTTCTTACAATGGATACTGAGTAAGCATGGTAACCCGGTCGCGTTCAAAACCAAGTCCCTTAGTTCTTTTCGTGGAGAGTGACAGGCGGGCACCGATCTGTTTTCCTGTACGAGATAGAGGATGGCGGTGTTTCGGCTGGAGAAGCTGTCCGCGATACTGAGATCCGAGCAGTGTATCAGAACAGAGGTGTGAGGGATATGCCTCATTTGTCCTTTGATGACGGTGTTCAGAAAGTTTCCTTCCATGAAGTGTAGCATTATGGCGTTGGGTCTCTCTTGCACGAAGGGCAGGCAGTAGAGCGGCGTGATGGGGGAGATTTCTGCGAGTGTGTCGAGCGAGTTGATCGCACTGTCGGTGATAGCCATGGGGAAGCAGCTCGAAATGACGTTGAAGAGCATTTTGGCGTTGGAGTGTCGGTCACCGCACAGGACAAGCGTGTTGAGTTCACCCTTGAGCCATGCACCGATGGTCGCGGATGCTACTCTGGCGTTGTAGCCTTCTTTTTCGAGGAGTTGGTAGAACCAGTTTTCCCTGGGGTTGTAGAAGGCGCTGGAAATAGGGTCGGACGCTTCGGGAGGCAGTTGGAGGAACAGGCTCTGTGACGGTCCGAAGCGCTCCCTGAGGACCGACAGGACGTATTCTCGCTCGTGGGCAGGCAGCAGATGCTTGGTGGGGAAGGTCCATTGGAGATCGTGCGCGATGCCCTCTTCGAAGAGCTGTTCGATGAGAACGGTGAGGGGATGCATGGGAGGGGCTTCTTGCGAAGAGTCTTCGAATAGGAAGAATTGATCTTCGTGCGAATCCTCTTCTCGTTTCGCACGCTTAGTAGTCGTCTGAAAGGGAGAGAAAATCAGCGTGATATTCGGAACATGTAGAGCACATATCACCAGCGGATGACTTGGAACATAGGTATCCGTACGGGTTGCAGCATTCGAGGACGTCGTTGGAGGTCGCGGCGTGAGTTTTGAGTCTCCGTATGTACCCGACGAGTTCAGCAGCCTCGAATGCGAGACCTACGGTGGGAGCTTCGAAGTTGATGATGACGTGCTGGTTTTTATTGGTGGGCAGTCGATCGGGGTGTGGGAGGGATCTGAGTCTAACTAAGCATTTGGTCGGCTTGATGGTGACTAATTCACCGTCGCTTGCGATGGTCGTCTCTCGTCCCTGGAGCATGTTGTTGACGGTCGGGCTTTGGAAAGGAAGGGAGTGGAAAGCTAGAGGAAAGTAGAGGAACTTGGTTTCGCGCTGCACCCTGGCGTATTCTTTCATGTCAAATTGATTCATGTCGCCCATGAGCACACACTCGAAAGCTCGTACGAGGGAGAAGGCGAATGGATCAGCGCAACTGGACGCGTCTCCGACTACGTACATAGTGTTAACGTAGTCTTTCCTGGGGTTGTAGGGGAATGCGTCTAGCCAGGAGGTGATGGCGTTCACCAGAGAGGTGAGATGCATTCCTTCTTTGGCAGCGATGGACGCCAGCTGCCTCGAGTTGGAAAGCGGCGAGTGACCATGTTTCGAGCGCAACTCTAGGGTTTGATAAAGACCGTAATTGCAATAGATGCGCTTGGCTTCGAGGAGCAGTTCGGCGGCGTTGTTGGCATGCCTTTTATACTGCTCGGCGTCGATTTCTTCCCACTGTGAGGGAGTGAAAATGGACCTGAGCTGCAGATGGAAGGCGAGCTCTGACATCTGTTCGTGATTGCTAGGCCTTTTTTGTCTCGTCTGGCGAGTCTGAGGGAGAAATGGTTTCGAGAACCGTTAGTTTACGACGCTTTTCCCAATGGGGTTTGTGATTATCTGTAAATCGCATGATTAGTTTAGCGGCTTTAAGGAAATTTTCCTGAATGTTTATATTGAGGGGTATGATATCATAGTTTCTGTCTAGAGTGCACCAGCGAAAGGAGTCGGAGACCGGGTCTGGGGTGTATACGATCCAGCTGAACTTTTGGTTCTGCTGGAGATAGGCAAAGTACGCTTTGAGGAGGACCATGATGTCCTTCGAAATGTTGTGCGCGAACGAATAGAGGAAGCGCGAGAACTGAAATTGGGGGATGTGACAGGACATGATATGAATTTTGGCATTGACTTTGAGAGTGGGGACGTTACCGATGGCCGTTCTGGGAGCCATGTTGTGGAGAACAACAAAGATGTAGAAAGCGGAGCAAGCAGCAGAGCGAGCGAAAAGCTTGGAGGGGAGCGCGTGAAAGAGCACCGAGACGCTAGAACCCGAACACAGTTTGTCCATGCATTCGTCCATCACGATAGCTAGAGGGCCCTTCTTCGAAGCCGTCACATAGATATTGTTGGGGTTCTCGATATTGAGGTTCTCCGGTGTAGTAGCTTCTTCGTAGGTCATCTCGATAAAATCCGGTCTGAACGTGCAAGTCCTGGGGGCGAATGTCCCGTCGTCCCTGCAGTCGTAGTTGGATTCCAGGAGCTGTAGGTTCCAAGCAGTTTGCTCGATAGGGGGGATCATATTCTTCTCCGGCGTGATAAAGATAACCGTTTCCGGGATAGGTTGCAGCATGTTGCACGAGATGAGTGCGCGCAGCAGATGACTTTTTCCGGATCCCGTTGGACCGTAGATGACGCCGATGACGGGTTGACGTCCCATGTTGAGCGACTGTAGTTGACCGTTTCTGAGATACTGCCTGTCGTGGTGTTCTTGTTGTTCGACGTTTTGTTGTAGTTCGTGGAACTTGGCGTCTGTTCCGCCGAGACTGTAGAACTCGTCGAACGGGAGAAACCGGTGCTCCTTGAAGAGCTGGGGCGCTAGATCGGTGGCTCCTGCGTACCATCCCGTGACTCGGTTGTAGAACTGTTCGCCGGAGAGGTAGTCTTCTTCTCGATACTGCCAGGCTTTTCTTTTCTTAGGGATCGCGCTCATCCGTCTCTCCGAGGAGCTCGAGTATTTCGTCACACTCTTCTGCCGTTAGGAAGGCATAGGGGTCTATTCGCAGCGGCGCAAGCGGTTCCTCGTCGCCCGGTACGGACACGTGGCGTACTTCCTCAACCGTGCGCGGGTTGGGGTGTTCGGTGTCGTAGGGGTAGAGGGCGTTACCGTACTGATATTGCGTGGGGTCCTTCCAGGGCCTCAGGATCCGCGTTAGCTGCTCGTTGTGGATCGTGAATGGCTCGTAGCGGCTGACCTTGTTAAGCAGCGTGGTCTTGAAGATGGTTCGCCTGGTGTGTAGTTCGGGAATGCGGCTGTCTGCTCCGAACAGTTGGTCTTCATAGCGCATCCAGCAACGTAGGAGGGTATCGTAAATAAGCTCCGCCTGTTTGTGACCCTTGGATCGAATTTTCCCGGTGCCCACATAGCCGCATTGAGGGTTCGTGCACACCGCGTCTTTGAGACCGTAGAGTTTTGGGGCGAGGAAAATCGCTTCGGAACTGTAGGTGTCGCTACCACACTGTTTGCACTTGATGTCACAATCACATGCCCAGTAGAGGTCTGGTTTCTCGGGGTCAAACGTGAGGCGTGTGCTCTTAGACTTGATGCGGTGAGCTCCTCGTGTCTTCATGCGTTCGTAGCCCGTCTCGCTGAGGAATAAGCTATCGGTATCCCCATAGAGCGTTTGCAGCTCTCGGTGGAGAATGTGTGTGCCCCTGTCCGGTCCGTGTAAAATTTCACACCATTCGCTAAAAAATGCTCGTGACCAGCCCAAAACAAAGCATGCTATTTGGGTGGCATAACGTTTGTTTTCGACTTGTTTGTCCAGGCTTTCTAGGTGGAGTACGGTGAGCGCCTCCGGTGGCGCGTCGAGGAATCGAATGGGCTTGAATGCGGTCTCGGTGGCTCGCGAGTGGGCGGGGTTTGTTTCTGCCGGCGGGCGCCCGTCATCTATATAAAGGCCGCAGGTGAGCGCTTCTTCCAGCTCCTGATCGACTTCGGAGAGGTCTGCCTCCTCGGCGGTAAGTACGACGTTTTCGTCCTTACTGGTACTCTCGCAGTGTTCGGTGTCCTCCTCCTCCTCGTCATCGCTCTCTTCTGGGGGTTTAAAATGTGCTAATAAGGATGGCGCAGAGAATGGATCTCCCGTCTCGTAAAGAGTTTTGCCCGAAAACGACCTGTCGTTGAGAAGCGTGACATGTTTGACCACTTGGGTGCCCTCGTAGATCTCCTTTTTGTCCTTGTCCGTGAGGTCTTGTTCGAACACGATACGCGTGGTATCCATGTTGGTCGCGAAGGCCCCGTAGAGGGCGTTGCTGAGCATTTTCGAGATGGATCTCATGACCTCATTTTTCTCTTGATCGGCTTTTTCCTTGGCCGCGATGTTCTTGCTGACGTAATCCGCACAAATGGTCTTCCACTCGGGGAAGACAATGTTCATGTCGTCATGCAGGGCGGTGACTTTCCACCCGCGGTTGTGAAGCGTGATGATGTCTAGGACGGTGACGACCTCGTCGTAGAGCACCTCGTTGGCCCATACGAGGCGACCGCCTCTGCGGTTGCACAGCGGAGGGAGGGTATCGAGCTGTTCGGGGGGCGGCGGGTAGGCTTCTATTTTGAGGATGGAAGGTTTGATGCGTGCGTCGAAGTAGCTGATGGGTGCGGGCTGGAGCAATATAGCGTTCAGCTCGTCCACGTGTGCCGCCGTGAACTTGGGATCGAGAGGCATACCGTGGGGCATGGGGTGGGTGAGGGCGGAGGCGTACATACCGCAGATGTCGAACACGTATACGGGTTGCGTGAACGGCCCTAGCACGCTGGGATAGCATCGACCCCCGCGCAGCGCCTGGCGAATGTACTTAAACATGGGTCGATGGGGAGCGTAAACCTCGGCCACGTAATCGGTGGGTATGGATTTGTTGGTTTTGTTGCCTTTTTTGTTTCGTTTCGCGGGTTTGGATTGTCTTTTGTCGAGCTGTTCCTGAACGTACTGCGAGAAGGTAAGTTGCTTCCAGAAGGCATGTGTATTGCTGGGGATGGTGGGCCTGACGAAGATGTTGTAATTGCCGTGCATGCCCAGCTCTTGCTTAAAGTATCGGTCGTAGCTTTCAAAGAGCGTGTGCGCCAGCTTTTGGGTGACGCGCACGTCCTGCATGCAGTACTCGAGACAGGCTCGTACGAGGTCGTACGGCTGTCCTGGGTGATTTTGGTTCCACAGTTGTTTTTGTTCATCGAGGACAGAGGCGTCCTCCCAGTAACGAGCGACGGGGAAGCCGTCGGCGTCCCGATCGTAGTGTCCGCGCGACACGTGTTCGTTGATGGCTTCGTACGGACAGTGTCCTTTGGACAGTTCTAAGGCGTAAGCGCTCGCCGCTTTGGCGAGTTTGGCTCCGCTGGTAAGCTGGAGTGTATCGCGCACCATGAAACGCACAAAGACGGACCGCATGTCGCGTTCGTCTACGACGCCTTTCACCCACCGGTGTAACCGGGTGGGGTCCTTTTTGCTGAAGTTGGGGTTGGGCATGTGGAATAGGATGTCGTTGAACAATAGTCTGCCGACGCGAGGCATGAAGGATCGATCGCAGCGGCAGGCGTCGGGGAAGAGGTCTCGTCGCTCGACCAGTTCGGTGGCGAGGAGCAGTTCGTCGAACTTGGTGATGTTGTGACCCAACACGATGACGTCTACGGAGTAGAAATCGGACGGTAAGGTGAGAGGCTTGCTGGGCTGTAGGAATTTTTCGTACGGGATGTGATAGATGGATGTGTAGTTTCCGTCGCTCATGAGTTCTCCGCAGAACTCTCGATTGGCTCGACAGTAGCGGCGCACGAGGTGTTCGGCGAAGTGCTGTTGGAGTCGTGTGCGGTAAGTCCGGAATCGTCGCGCGACCTCGCCCGGCTTCGGGTCGATCCAGTAGAAGCCTTCGTCGAGCTGTCGGACGTCGGTGTCTTCGAGCGCTATTTTGCGCGCGACCTCGACGAGCGCGGGGTCTCCGCTGAGCATGAAACAGAGCATGAACGGTTGCATGCGCTTCCCCTTTTGTTCGAAGACGGTGTACGTTTCGATGTCGTAAGTGAGAAAGAGCTGTTTGCAGTGGGGGCTTTGCGCCGGGCACGAGAAATGGACGTGTTGCCACATTTCGCTACCTGTTTTCTGTACGGCGTGGTAGTAGAAGGCGGATCGCCTTTCGTTGCACGCGTGGTTTCGCACCCAGTGTCGCCCGCAGGTGGGACAGTGTTGGACGGCGGTTCGCGAGAGGACCCAGAGCCAGCGTTCGGGCTCTCGACGGGCGTCCCGAGCTAGCAGGATGGGAGGTAGGAGCGGTGCTTCGTCCTCGAAGAGGCGTTCGATGTCGGCGTTTCTTCCGAGGAACTTGAGGACGCCGATGTACTTCGGCCGGAAGACGCGCAGGGAGTTCGTCGGGTCACGCTCGTAGAAGGCGTAGTCGATAATGTCGTACTGGCGACTGCGTTCGGTAAGGAGGAACTTGTGCAGCTTGAGGAATTTTTTCATGCCCCGCGCGAAGGGCACGCATTGTAATTTGAACGGCTCTCCTTCGATGTAGAAAATGCCATTTTTTAACACGTCTCCGGGTACGGTTTTGAACAGGGTCGATGGGGGGCGCCCGGGTGCGCTGTCGGGATCGTTCCGCTGACGCCTGGGTACCGTTCGCACGAGGTGAGAGTGCTCGCCCAGGATGGAGCGTCGCAGACGTCCCGCTGCGACGTGTTCCATGACGCTAGCGGCGCTGTTGGGGATGGTATCGCTGTTGGAAGTCGCGCTCTTCCCTCCTGTTTTGTAGGACTTGGCGTAAGTTGTCGGTAATGCGCTGATTGGTGGAGATGGCTACGATGCCCCGGTACTTGAGTCGGAAGCTGATGTCGATGCTCTCGATGAGCTCCTCGCTAAGGTTGAGTTGCTTGAGGACCTCGTCGATGTCTCCCGACTTGTCTCTGTATTGGATATCGGAGAGAAAGAGTTGTTGATCAGTTTCGTCCATGCCCTCGAATTGTCCCGTGCGCTCGACCATTAGCAGGAAGTCGCGGAGGATGCGGTTCCATAGGGTTTCGAAAATGCGCGACGAGTTGGACTGCTCGCTCCAAATTCGTTTAAAAACCTGCTGCGCGTTGACATCCCAACCCACGATGAGCACTTGCAGCGTGTCGATTTCCACGTAGCGCCTGAACTCGCGGTAGTTGATAAAGTGACTGTACAGGTAGTATAGCGTGGAGGCGATGTGCTCCGCGAGGAAGAAGTAGAGGACCCACTTCCTGAGGAAGGAGTCGGTGACGAGAGCCACGTCGGATTGCTGTGTCTGCAGGAGCAGACGGTAGAAGGCGGTGGCGAACTGGAACAGGTCGTGCCTCTGCGCGGTGCGACTGAGCTCTTGCTGTAAGGCGTCGATGGCCTCGAGCGCGGTCCGGACGACCTCGTCGAGGAGCTGTTCTTCCTCTTCCTCTTCTTCGGGCGGGAAGGGCTCCAGTTCCGGGAGGTATTCCTCGGGGGGTTGGGGGGAGGGGGGTCTAGGGCGCCGTCGGCGACGCGTGAGCCTCGGCAACCTGTCGACGAAGCGCTCTACCGCCCTCCGTCGGATCTGGCGGATCTGGGACGCGGTGATGGCGCGTCCGTGACGGTCGCGAGGCCGTAATCCGGATCTGTCGGTGACTCTGCGGTTTCGCAAGGTAATGGCGCCTCCCGAGAGCGAGGTGTCGCTAGCCAGAGTACTAACGAGACATCTCGCCATTATGGACGCAGGATCCTGCCCGAGCCATCCCGCTTGAGGATCGTTTTCGACCGCGCGGACGAGCCGCTGAGTGTCTAGCTCGCCAAAGGCTTCGACGAAGAGGTTGAGCCAATCGTCTTCAGCGAACACTTCCGATCCAGGTAAGAATCTATATGTGCTTTCGGTGGTAAAGAGGTCGTAGGCGTTGCAAAATAGGTAGTGACACAGCGCAACGCGGAGGTAACGGATGGCCGCGAGGATCGCGGCGTCGCGATGCGAAGCCGTGCGAAGGACGACGTCGTCCCTGAGCCCGCTCGCGCCGCTACCCGTCAGATAGCTGTGATCGTCGGCGTTGAAAGGTTGCGGAATGACCCGTCCGTCTCGACCCACGACGACTCCTCTGCCTCGGAGATACGCTCCCTGCATGTCGGCGGCGATGCGATCCATGAGCACCGCGTTGTGCATCTGGGTGAAGGTGCCGTGAAAGTTGTCTAGGTCGAGGAAGCGCATGTACTGTCCGACGCTGACAGAGTACGAGCAGTCGGTGAGGCAGGTCCAGAAGAGCCTGCGCGGTCGTTGTGTCGGAGGGCTCTCGAATCCGAGCTGCATGAAGACGCGGTTGTCAAAGAAGTAGTTGTTGAGCGTCCTGTGCATGTACTGGTAGCCGAGCAACAGATGCGGGGGCGGCAGTCCGTTGTAGGGGTTCTGCGCGACGTTGGCTCCCCCCGGCGTTAGGTCGCGCAGCTGCATGAGCCTGTAGTCGTAGATTCGGCTGACGAGAAAGACGCTACGGGGATGCACGAGGGCTGGTTGCGTGCGCACCATCGGGAAATCGGCGGCGATGACGGGTTCGCAGAAGCGTACGGTGTTTAGGCTTTGGCCGGTGAGCTCCGCGAAGATCCTGTAAGCCTGAAATCGACCCCTGACGTTTTTAGACACGCGCGTGGCTGCATAATGCACCCGGTTCTTCAAAACGTGCGAAACGCGAGTTTGGGTTCCGGCGGAAGATCGAGTCAGTCGCAGCAGCACCAGCAGCAGCAGGAGTTGCCGCCCGTCTACGATCAGCAGCGTCAAGCCTACCAGCACCAGCAGCCGTACCAGGACCGCAGCGCGGGCGGGGGTGGCGGCGCGCGAGCTCCCCCCGACCCTCCCCGATACCCGGCACAGCACGCGCTTCCGGTGGCGACGGGGCCTCCGGAGATGGCGGCGGGGGCCGTGCCAGAGGAGCCTCCGTCGTGTGGGATGGCGGTGGGAGCCACGCTAGACCCGACCCGCATGTACGAACGTGACGCGGCTAGAAAGGGGGCCATTCCCGAGGTGAACCTGTTTAAGGCGAAGCCGGACACGGTTCCTCAAGGCGATTACGATCGGGACATGATGTATCGCTCGGGACAGGTAGTGCAGTTGGACAGGAACCGGGTGCTGCGCCCCGAAGACTTCGCGGCGGACGCGGGTGACCCGACGTTCTCGCCCGCGGTCAATCACATGAAGGCCGCCGAGTTGAAGCGCGCCTCGGAGCAAACGGCTTTCGGGGAGGAGATGCGCAACGTGTGTCACCAGACGCGTATCCGCACGGCGCTCTCCCGACCGGAGGTCGGCGCGGGCATCTACTACCTGTACGATTTTGTGCAGACGTACATGGAGCACCCGGACGGGCGCGTCAAACTGAACCCGCAGCTGGTGCTGGTGGCGCAGCACGCGGGAAATACGTCGCTCGCGCAACGCCTATGGGCGATCGCGGAAGAGAAAAACGCGTGGCTGAGAGACCTGATAGAGATGGCGTACATGATCGTGACCGACCCGTACCTGAGCATAGAGCAGCAGGTATCGGCGGTGTGCACGACGGTGGTCGAGCTGAGCATGAAGTACGCCAAGTTGGCCGCGAAGAACGGCTACCCGTCCATGGCTCAGATGGCCAAGGCCCAGGAATTTTTCTACCGGGTGATGCAGGCGGTGCTGGACTTGGGGGTGCAGTTAGGCGTGTACAACAACAGGCCGGTAACCTTCCGTCAGAAGCGGATGAGCGAGATTCCGCAGATGACTGACGCCGAGTACATGTTCGGTTTGACCCAGGCGCTCGAGAACCGACCTCCCCAAGGGGAGTTTCCGGCCGACGGAGAGTTTTCCGACAGCGGAGAGGAGGATGAGTTCGACTGAAGTCTTCGGCGCGCTGGCGCCGGTGGGGCGTACTGAGGTGGCCGACGCGCTGAGCTCCCACGCCAACAGCAAGGATGCCCGCAGTCTCCGTTACGAACCGTACGCTAACCGCCTGATCAAATTGCAAACGGCGATGGTGCCCCCTAAAGTGGACGGGACTTCGGAACGGGTAGCGGAGGTGGTCAAGGGCTTGGCAGAGCAGGGCGCCATCTACCCCGATCAGATGGGAGCGATCCATTCGGACTTGCTGAACCGCGTGTACACGTGGAACTCTATGGGCGTTCAGGAGAGCATTCAAGCTCTGGTGAACGACGTCATCCACGGTCAAAATAAAGTGTTGCAGGACGAACTGGCACGCACGCGCGAGATCGCGAACGCTTCCATGTTGACTCGTTTCTTCGACAGTCTGTATAAGACGGTCGACCGGGGGCAACGCAACTTTGAGGGCTTCAAGAAGTTGCTCCGCTTGTTCGTGAATAACGTTCCGAACGCGGAAGTGTACAGCTCGGGCGGCTCCTTCAGTTTGCAGATTAACATGGGGGGTCAGAGTCAGAACATCAATCTGACGAACGCCTTCGACAACCTGAAGGACATCTGGGGGGCGCGGTGGGATGCCGTAAACAATCCGCGCATAGGGGCGCTGCTGACGCCTAACACGCGCGCCCTGCTCTTCTTCGTGAGCACCTTCTACGACTACGGCTCGATGGAGCCGGGTAGCTACCTCGATAACCTGATGCGTCTGTACAAGGAGGCCATTAGGGCCGACACGGACGCGGAGGGCGACGCCATCATGGAACTCGGCGACGCGGGCGCCAATCTGAATCTCAAGTTTAACCAGTACAAGGACACGCTCAACTATCTGCTGCAGAATAAGCCGTCCATCCCGCAGACGGGGCCGCTAGAGATGAGCCCCGAGCAGGAGAGTCTTTTTAAGTATTTGATGCGTCAGCTGAGACGGGCGTTGAAAGACGGCGTCAACTCGGACATAGCCATCAGCACGATGGCGCAGTACGTGGATCCGCGTTTGTATACGAGCAATAAGGTCTTCATCGACAAGCTCCAGAACTACTTGCTGATGGCGAGCGCGCGCAATCCCTACTATTACAAGACGATCGTGCTGGATCCTCACTGGGTGCCGCCAGCGGGCCTTTATACGGACAACTTTGTGATCCCGGAAATGATGCCCAATTTTAGTGACTTTGCGAGCGAGCTCGAGTACGGCGGTCCCTCGCGGGACGAGTACTTTGACGACAGTCCGTTCCGTCCACCGCCCCAGAAAAAGTTTACGGAGAAAGAGCAGGCCGACTACGATTCGCTGATCAACTTTTTCGACTCGACTTTGGGCGTGCAGTCGGAGGCCGGTTGGATTGCAGATCACCGCCTCCCGCAGGCCTTCGACGGCGCGCTGAACGTGTCCGAGCGCACTCCCTACAACACCCCCCTGCCCGACGACGCGCCGATGCGCAGTCGCAACGCCTCGGTGAGCTCGGCGACGGACGCCTTAGGGCAGCTGAAGCTGAGCGGCACCGGGGGCGCCGGCTTCTTCGACAGCCTGAAGCCGAGCGTGGGCACACGCCGTTCGACAGGTCTGGCCAAGGGACTTGCGGGCACGGGACAACCGCCTTGCCCATGGCCGGCTAGCGCCGGCTACGCGTCCGCGGGCTACGGGCCCGCTCGCGGAATCAGAGGGTCGGGACTGGCTAGACGAGCGTTAGCAGCGAGGGGTCTCCGTCAAGGCAAGCGCCTACGCTTTTACTAACGAGAGCTAGATACCATTATTAAGGGACACTTACCGCCATTTCGACAGGATCTCCGACAAGGAGTGGTGCAATGTGGGGGTTGCAGCCGCCGACGTCGATTCCGCCGCCTCCTCCGCCGACCGAGTTAACGCCCTCGACCTATCCGGCGATGGTGAACGGCTATCCGCCTCCGGCCGCGTCCGCGCAGAGCTGTCCCTCTAGCGACGGTCAGAGCGAGCTGTATATGCCCCTTCAGCGGGTGATGGCCCCTACGGGGGGACGGAACAGCATTAAGTATCGCGATTACACGCCGTGTCGTAACACCACCAAGCTGTTTTACGTAGACAACAAGGCTAGCGATATCGATACGTATAACAAAGACGCCAACCATAGCAATTTCCGCACCACGGTGATCCATAACCAGGATCTGGACGCGGACACGGCCGCCACCGAGTCCATCCAGTTGGACAACCGCTCCTGCTGGGGCGGCGACCTAAAAACAGCCGTGCGCACCAACTGCCCGAACGTGAGCAGTTTTTTCCAGAGTAACAGCGTGCGCGTGCGCATGATGTGGAAGCGCGACCCGCCGACTAGCACGGCTCCTCCGAGCGCGGTAGGCAGCGGCTATTCGGTGCCCGGCGCGCAGTACAAGTGGTACGACCTGACGATACCCGAGGGTAACTACGCGCTGTGCGAACTGATAGACCTGCTCAACGAGGGCATCGTGCAGCTCTACCTGAGCGAGGGGCGCCAGAACAACGTGCAAAAATCGGACATCGGGGTCAAGTTCGACACGCGCAACTTCGGCTTGCTCCGCGACCCCGTGACGGGACTGGTAACTCCGGGCACGTACGTGTACAAGGGTTACCACCCCGACATCGTGCTGCTGCCCGGATGCGCGATCGACTTTACGTACAGCCGCCTGAGCCTGCTCCTGGGCATAGGGAAGCGCGAGCCCTACTCGAAGGGGTTCGTTATTACCTACGAGGATCTGCAGGGAGGGGATATCCCGGCTCTGCTGGACCTCGACTCCGTCGACGTGAACGACGCTGACGGTGAAGTGATCGAGCTCGACAACGCTGCTCCCCTTTTACATGACAGCGCGGGCGTGTCGTATAACGTCATTTACGACCAGGTGACGGGTAAACCCGTGACGGTGTATCGATCGTGGATGTTGGCTTACAACGTGCCTAACTCGCCGGCCAATCAGACGACCTTGCTGACGGTGCCCGATATGGCGGGCGGGATCGGGGCGATGTACACGTCCCTGCCCGATACCTTTATCGCGCCTACCGGGTTCAAGGAAGATAACACGACCAACCTTTGCCCGGTCGTCGGCATGAACCTGTTCCCCACCTACAATAAAGTTTATTACCAGGCGGCGTCCACGTACGTGCAGCGCCTGGAAAATTCCTGCCAGTCGGCCACAGCCGCCTTCAACCGCTTTCCCGAAAACGAGATTCTGAAGCAAGCGCCCCCCATGAATGTTTCGTCCGTGTGCGATAACCAACCCGCCGTCGTTCAGCAGGGTGTGTTGCCTGTGAAGACCTCGCTCCCCGGACTGCAGCGCGTGCTGATCACAGACGACCAGCGTCGTCCGATACCCTACGTGTATAAGTCTATCGCGACGGTTCAGCCGACCGTTCTGAGTTCCGCGACCTTGCAGTAGGACTGAACATGTCCATTCTGATATCCCCGAATAACAACACAGGTTGGGGTATGCGTCGCCGCTCTAGATCATCATCCATGCGCGGGGTGGGGATGCGTCGCAGGGCTCGCCCTCTGACGCTGCGCTCGCTCCTGGGTCTGGGCACCCGGAGGAGACGCGGCTCCCGCCGCTCCCGGCCGAGGACCACCAGCCGGCTGGTCGTCGTGCGCACCCGCACCAGCAGCATGCGAAGACGTCGTTGATCGTCGCAGCGCAGACTACGTCGCCTACCGTCGCCTCCATCTACCGATTCTAGTTCGAACGACTTGTTATAGCCTTCGTGACTTTGGTAACGACTTTGTACCGTCCCTGTACAGACAGGACGGACCAGCGCCGCCTTCGTCGTCCCCCGACGCGTCGCTAATTCTTCGACCATGCCCGCCGTGCTTTTGACCGGGGGTCGCGCCGCCTCCAAGCGTAAATTCAGCACCAAGCAGCGTCGCAAGAAAGCGGTGTCCGTGCCCAAGATTCGCTCGCGCAGCGGCAAGCGCAGCGGCGTTCGGAAGCGTTCGTCCATTTCGGTGCCCGTGAGCGGCACGGCCAGCGCCTCGGAGAGAGCTGCCTTGCAAAATCTGGCGCAACGCCTTCAGAGGGGCAACTACACGGCCTGGCGCTCGGCGGACCCCTCGGTCGCCGCGAGCGAAGCTGCCAAGGCGGCCGCCGCCAGCGGCGCCGCGGCCTACGTGCGAGACCTGACTACGGGCACCGCGGCCGAGGCGGTCCCGCTCACCGGCACCGGGAGGCGGCGTCGCACCGGGGCGAGGCGATCGATGCGCGGTGGCTTTTTCCCGGCTCTGATTCCTCTGATCGCCGCCGCCATCGGCGCCATCCCCGGCATCGCCGGCACCGCCGTGGGCATCGCGAGCCTTAAGGAACAGCAGAGACAATTCAATAAGTTGTATGGCAACAAGTGAGAGAGAGAGAGAGAACCGAAGTGCTGACTGTGTGACTGTCGTCTAAGACGTTTCCAATAAAAATTTTGTAGACGATCGATCACTCGCCGCCGTCGTTATGGACTACGCCGCCTTGTCGCCTCACGTCGGGTCCTGGGCCCTGAGAGAACATCACCTGGGAACCTCCACCCTGCGCGGGGGTGCCATAAACTGGTCCAACGTGGGCTCGCGGCTCTCGAGCGCGCTGAGCTCCACCGGACGATGGCTGTACAACACCGGCAACCGCTTCGTCCACTCCAATGCTTTTAACCAGATAAAGCAGGGCCTTAAGGATAGCGGCATAGTGCGCAACGTGGCGTCGCTGGCCGGTGAGACGCTCGGCGCCCTGACGGACATCGGGCGCCTGAAGCTGCAACAAGATCTAGAGAAGCTTCGCCGTAAGGCTCTCGGGGAGGAAGGTCCCGCCACGCAAGCGGAGCTCCAAAGCCTGATCCAGGCTCTCCAGGCCCAACTCGCCGCCGGAGCCGAGGTCTCGCCGCAGGGTTCTGCGCACGTCCCGCAGACGGTACCGGCGCCGCCCGTGCCTACCACACGCCCGATTCCCGAGATGGTGACGGAGGTGAACCCTCCCATCACGTCCTCCGCTCCCGCCGTGCCCGTGGTGGACGTCCCGACTACCCTAGAGATGCCGCCGCCCGCGAAGCGAAGGCGAAAGCGAGCCAGAGCGGGCTCCTGGAGAGCGAGGCTCAACACCTTGTCGGGCACCGGAGTGAATGTCAGCAGTAGGCGATTGTGTTACTAAACGGGTTGTGTATGTATGTCGCGTTTCGTCTAGGTTCGCACCGCCATGGCGGCCCTCACGCCCGACCTGACTACCGCGACTCCGCGGCTCCAGTATTTTCACATCGCGGGCCCCGGGACGCGCGAATACCTCTCTGAGGACCTCCAACAGTTCATTTCCGCCACCGGAAGCTACTTTGACTTGAAAAACAAGTTCAGACAGACGGTCGTGGCGCCCACCCGAAATGTCACGACAGAAAAGGCTCAACGGCTGCAAATCCGCTTTTACCCCATCCAAACCGACGACACGTCGACGGGCTACCGCGTGCGGTACAACATCAATGTGGGCGACGGTTGGGTCCTGGACATGGGGTCGACCTATTTCGACATCAAGGGAATCCTAGACCGAGGGCCGTCCTTCAAGCCCTACTGCGGCACGGCTTACAACCCGCTGGCTCCCAAGGAGTCCATGTTTAACAACTGGTCGGAGACGGCACCCGGGCAGAACGTGTCCGCCTCCGGTCAGCTGTCCAACGTCTATACCAACACGAGCACCTCCAAAGACACGACGGCGGCGCAGGTGACGAAGATTTCCGGCGTCTTCCCCAATCCCAACCAGGGACCCGGAAGAAATCCTCTGCGACGGGTACAAAACGCCAACACCGGCGTGCTCGGTCGCTTCGCCAAGTCTCAGTACAATTACGCTTACGGTGCCTACGTCAAGCCCGTCGCCGCCGACGGTTCCCAGTCCCTCACGCAGACCCCCTACTGGATCATGGATAACACGGGCACCAATTACCTGGGAGCGGTGGCCGTCGAGGACTACACCAACAGCCTCTCGTACCCAGATACCATAGTCGTGCCGCCTCCCGAGGACTACGACGATTATAACATAGGCACCACGCGTGCGCTCAGGCCCAACTACATCGGGTTCAGGGATAACTTCATTAACCTGCTGTATCACGACTCCGGCGTGTGCTCGGGCACCCTCAACTCGGAGCGTTCGGGCATGAACGTGGTGGTCGAGCTGCCCGACCGGAATACCGAGCTCAGCTACCAGTACATGCTGGCCGACATGATGTCCCGCCATCACTATTTCGCCCTGTGGAACCAGGCCGTGGACCAGTACGACCCCGAGGTGCGAGTCTTCTCCAATGACGGTTACGAAGAAGGCGCGCCCAGCTACGCCTTCAACCCCGAAGCGGTAGGCGCGGGAGAAGGCTACGGCCCCGATCTCAGTCAAATTAAACTCTACACCAACAACACCGCCGCGAACGACAAAAACACCGCCGTGGCTAACGCCACTACCAACTTCTACTTCGGCACGGTACCCTCCTACGAAATCGATATCAGCGCTACCCAGAGGCGCAACTTTATCATGGCCAACATCGCCGAGTATCTGCCCGACCGTTACAAGTTTAGCATCTCCGGCTTCGACGCCACCAGCGTCGCGCCTACCACCTACGAGTACATGAACAAGCGCGTCCCCCTCACCAACGTCGTCGACATGTTCACGAACGTGGGTGCGCGTTGGTCCATCGACCAGATGGACAACGTCAACCCCTTCAACCACCACAGAAACTGGGGGCTGAAATACCGCTCCCAGCTGCTGGGAAACAGTCGCTACGTCAACTTCCACATCCAAGTGCCCCAAAAATTCTTCGCCATCAAAAACCTGCTGCTGCTCTCCGGCTCGTACACCTACGAGTGGGTGCTGCGCAAAGACCCCAACATGATCCTCCAATCCAGTCTGGGCAACGACCTGCGCGCCGACGGCGCCAGCATCATCTACAACGAGGTGAACCTCATGGCCAACTTCATGCCCATGGATCACAACACCAGTAACCAGCTCGAGCTGATGCTGAGAAACGCCACCAACGATCAGACCTTCGTGGACTACCTGGGAGCCAAAAACGCTCTATACTCGGTGCCCGCGGGCTCCACCGCCCTCACCATCAACATTCCCGCTCGCACCTGGGAGGGGATGCGCGGGTGGTCCTTCACTCGCATCAAGGCGGCCGAGACGCCTCAGCTGGGCGCCCAGTACGACGTCAACTTCAAGTACTCGGGCAGCATCGCCTACTCAGATGGAGGCTTCTACCTCTCGCACACCTTCCGTAACATGAGCATCCTCTTCGACACGTCCATCAACTGGCCGGGCAACGACCGGTTGCTCACGCCTAACATGTTCGAGATCAAGCGCTCGGTGGCGCTCGACACCGAGGGCTTCACCATGAGCCAGTGCGACATCACCAAGGACTGGTACCTGATCCAGATGGCCACGAACTACAACTTCGTCTATAACGGCTATCGATTCTGGCCCGATCGTCAGTACTTCCACTACGACTTCCTGCGAAATTTCGACCCCATGACGCGCCAGGGACCCAACTTCGCATTGCCCGGCCTCTTCGACCTCGTGTCTTACACCCCTACCACGGACAACAGCGGACAGCAGCCTAGTCAGGAAGCCGTGCGCAACAACTCTGGGTTTATCGCCCCCCGCTCCTGGCCCGTCTGGAGCGCTCACCAGGGCGAGAGCTGGCCCGCCAACTGGCCGTACCCGCTCTGCGGTCAGCAGGCCATCCAACCCGCACAGGTCCTCAGCTACAAGAAGTTCCTCTGCGACAACTACCTGTGGACCATCCCGTTCAGTTCCGACTTTATGTACATGGGCGAACTGACAGATCTGGGCCAGAACCCCATGTACACCAACAACTCGCACAGCATGGTCATCAACTTCGAGCTCGACCCCATGGATGATCCCACTTACGTGTACATGCTCTATGGCGTGTTCGACACCGTTAGGGTCAACCAGCCCGAACGTAACGTGCTAGCTATGGCTTACTTCCGTACGCCTTTCGCCACAGGCAACGCCGTGTAAACCCTTAGAGCGTCGGCATGACGGGGACCACGGAGTCTCAGTTGCGGGACCTGGTGGCAGCGATGCATCCTCGTCACCGCTTTCTGGGCGTGTTCGATCGAACCTTCCCCGGATTTCTGGACCCGGAACGCCCCGCGTCGGCTATCGTCAACACCGGCTCCCGGTCCTCTGGCGGCATGCACTGGATCGGGTTCGCGTACGACCCGCAGTACCGGCGCTGTTATATGTTCGACCCCTTCGGGTGGTCCGACAAGAAACTGTTGGAGTTATACAAAGTTAAATACGACGCGATGCTGAAGGCCACCGGCCTGAGCCAGCAAGACCGCTGCATCGAGCTGGTGCGCTCCGCGCAAGCCGTGCAGTGCCCGTGCTCGGGCGCCTGCGGGCTTTTCAGCGCGCTCTTTATCGCCTCTTTCGATCGCTACCGACGGAGTCCGATGAACGGAAACCCCATCATCGACACCGTGGTTGGCGTCAACCACGAGAATATGTACAAACCGGCCTTTCGCGAGATCCTGCACCGGAACCAGGAGCGCATGAACGCGTGGTTCGCGCGGAATAATCCCTATTTCCAGCGTCACGCCGAGCTCCTGAAACGCGAAACGGCAATAAACACGTTACCACAGAATCACGTACAACAAGCATAGCGACTCCTTTATTGTGAAAGGAAGGCAATAAACATTCGTCACTCTGAACGAACGTCTCTCTCTCTCTTGTTGCGTGCGTGCGTGCGTGCATGAATGCGTGCTCCTCTCAAGCCCGCGTAAGGCACTACGCGAATGGATCGGAATGGGACACGGGACAGACGGGAGCGATGACTTCCGTTTTGTAGGCGTACTTGTCGTTCCAGCGGAACTCGCGGACCTGCGTGGCCGCCTCCGTGCCCAGCGCGGTGGTGATCAGCTCGTTCGCGAAAACGTACGCGTAGCGGAGATCCATGTAGGAAATGCGCCACGAGCAGCTCTTCTCGGTGCGCCTCTGCGCGCGGCTCGAACCCGCGGGGTTGGAACCGCCGGGCGCCTGCGGGTTGCAACAGGTGTACACCATCGTGTGCGGGTGCTTGTGGTGCGCCTTCATGTCGGCGCGACTCTCCAGCATATCCTTGGTGATATCGTCGGTGCCGCTCAGCTTGTAGGGGGTCATACGGCATATCTGCCGTCCGCTGATGGGAGCTTCGCAACCGTAATTGCAGTTGCAGTTGGTCGAGATCAACACGCACTGCTCGATGCGCGAGCGATCCGCGTTCGGGTACAGCGCCATGGTCCAACTCAGGTCGTGCTTCATGGCGCTCAGAGCCTTCTGCGCGTCCGAAAAGACCATCGCGCAGCTCCCGGTCGCGTGCGGGTACGGGAAGCCGTTGTGCTCCTTGTCTTTCGCGCATACCGCGTTGTTGTCGAAGCGGAGCACCACCACCTGCCGCCCGAAGCGGTTCTTCTCCACGCGACCGCCCTGCTCGGCGATGGCGCGCTTGCCGGCTTCGCTGGTCGGATTCAACTCCACCGTGCGAGGCTTAAGGCTCATCGGAATGCCGTGAAAGCACTTCGGCATGGTGGCGCCCTTCCAGCCGTGTCGCCAAACGTGCGCGCCTCCCGGTACGAACTTCGGTTCCAACCCGGCTAAGTTGTAAATAATGGCGGCCAAAAACCTGCCCAACTGCCCGTAGAAGGAATCGAAGCTGGAGAAGGTGAGTCGGAACTCGGGGTGCCGTCGGCGCATAAAGAGACCGGCGACCTTGGTCCAAATGGCGTCCAAGGGCTCGATGGTGCCTCCCTGCCAGCGCATATCGAGAGATTCGCACACGGTCGTCAGGTAGGCCATGGCCTTTTGCGCGCTGAAAGTGACGGGATCCTCCGCGAGGGCGCCCGCCATCGGGCCGCCGGTAGACTGGAACTCTTCGGCGTCGCTTTTGAGCTCCTGCTCCGCTTCGAGAACATCGCTATCCGCGAGCTCGGCGGTGCTGGTCGTCTTCTTCTTCGGCGGAGGGCGCTTGCCGCGCTGCGGCTTGGCGGGCGGCTTCAGTTCCGCCATTAATTCCAGCTCGGAATCCGAGTCGTTCAGGCACGCGGCCTGGTGTTTTCGCTTCTCGCCAGGCATTTCGAGGGAGCGCGAGCGTGAGCGGTGAGACGTGGACGGTCGTTCTACGGCGTCGTCCTCTGAACTTGAAAAGCCCGGGTCGTTAGCTATAGACCTTTTCAGGTCGGTCTTCCGACCTATAGGAAAGCGGACACGCCCAAATCCGTTAGCTCCTCCCATCGTAGCAGTGGCGGAGAAATTAAACCCGATGTTGCGCACTACGCGCTTAGTCGCCTCCTACCTCGCGGACCGCGGATCGTCGGCATCTTCCTCTTCTGCGGCGAACGGTAAGTTCGAACATCCGACTCCGCTTCCCTCTCGTCCTCCGAGCTGAACGTCTTCGTTAGCGGCTCTCTGTAGGCCGCGTCCCTCTGGAGCTTCTTTTTCCTCTTGGTCGGTGAGCCGTACGACCGGCTCGTCGAGAGTCTCGCGCTTCTCTCGCGCTCCTCCTGCTCTTCCGCGTCGGAGGGCATCGGGTCCTCGTAGTCGCTGTGCTCGCTTCGGCTTGAATCTATCTCGACTTCCATTGCTCTCTAGGGGACTAAGGCGCACATAATCATGATGTCGAATCCGTCGGGTTACGGACAGCTGAAAAGCTTGGCGACAGTGGGCTTAGTGCTGCGCAGCGCCCTCGAGCGGTTTCCGTGGACCGATTACGTAAGTCATCTTCGCGACCACGTTAGTACCACCTATCGCAAAGAGCTGCCCTCTAGCGAGGAGTTAGTCGAGATCGAGCTGGACACCCTGGCCGAGATACTCATTGACCGACTGGGCCAAGAAACGGCGGTGCTTAGCGCTTACAAAGTCTTGGAAGACCTTATCGAACGCGATAAGGAAGCGCCAAAAGAGGAAGCGGAGGCGCCGAGTGGGAAAGTACCTAAACTCCCGCTGGACCTTCCCTCCATTGTTCCCGAGGAGAACAAAAGCCCCGAGGCTGACGTCCGAAAAGACGTGGGCGAGATGGAAAGCACCGCCGACGGGGATAAAGCCCGTGGCGAGGAGCCCGCAGCTGAGCGCGAGGCCAGCGACACCGCCGGCGCCGACGGCGAGTTCCCCGCGCCGGAAGACGAGCATCCGGACGATGGGGAACCGGATGAACCGGCCGACAGAGACGACCGATCGGGCGAATCGGACGCGGATAGCGGTTACTATTCGGCAGATGGGGGACGCGATGCAGGCTACGACGGAGAGGCCGCTCGACCCGACACCCCTACGGACGAGTCTAGCGCGCCGACTACTCCATCCACAGCAGTGCGACGCTCATCGGGCGAGTCTAGCCCCGATCGCGGTGGCTGCTTTAGCCACTCTAGCGACTCTGAGCTCGGCTGTGCTACTGAGACTCGCGATCCGTTTGCTGCAGGGCTGCGCAAGTGCATCGAAAGGCAAGCCATGATCCTAACGGGAGCCCTCAAAGACGCGCAGCTCGACCCGCCCCTCGACAGCATGCCACTTACCGTAGACGCGGTGCAGAGACAGTTAGAGCGCTTTCTCTTCAACCCCGACCCGAAAGTGCCGCGCGAGCACGTAGAGGCTCGCTACAACTTTTATCCGCCCTTCATGACGCCTAAAGCCATCGCAAACTACCACATCTTTGCGGTAACCGCCCCCATCCCGCCTAGCTGCAAGGCCAACCGGAGCGGATCCGAGGTGCTCCGTGCCGCGGAGAACGCTCGCTTCTTCAAACGCTTACCTCGCTGGAAGCAGGGCGTGACGGTCGACGACGGTCTGGGAGACGAGGTGTCGCCTATAACAGAGCTGAAAGACGCCAAATTAGTGCCGTTGCGCGATGACACCTCCCGTCTCGAGTGGGCCAAAATGCGCGGCGAACACGTACGCTATTTTTGCTACCCCTCCCTCCACATGCCTCCCAAAATATCCCGCATGCTCATGGAGGTACTGCTCCAGCCATTCGCTCAAGAGGTAGCGAGCGGTGGCGAGCAAGAAGACCCCGAGCCCGTCGTTTCCGACGCGGAACTGGCGTGCATCGTCGATCCGGAGGGCGTGATGCAACCACACGCGCTAGCTAGAGCGATAGAGGTCAGACGGCGCATGGTAGCGCAGGCCGTCCGCTATACCGCTCAGCTAGAGCTTATGGAACGCGTATTCCGCGAGCCTTCCTCGATCAAAAAGGCACAAGAAGTGCTCCATCACACCTTCCACCACGGTTTCGTGGCGCTCATTCGGGAAACCGCCAAAGTCAATCTAAGCAACTATGCCACCTTCCACGGGATCACGTACAACGACCCGCTCAACAACTGCATGCTAGCCAAGTTGATGGAAGGCTCGGACAAGCGAGATTACGTGGTGGACAGCATCTACCTCTTCTTGGTGCTCACGTGGCAAACGGCTATGGGCATGTGGCAGCAAGCCATCCAGGAGGAGACCATCGAGGCTTATCGGGAGGCCTTTACTCGGCTCCGAAGAGCTATTTACGCTCTCGAAACACCCACCGAGATCTCCAAAGCCATCGTAGACGTGCTCATGGACGGAGACCGACTGTGCGCCGAAATGCGCAAAGCTCTCCCCAACTTCACCAATGGCAGCCAAATCAGCGCCTTTAGGCAGTTTATCATGGAGCGCAGTAACATTCCCACCACGGCCGCCCCCTTCCTACCCTCCGACTTTGTGCCGCTCTCCTTCCGACAAGCCCAGCCTCTGCTCTGGGACCAGGTGTACCTCCTCCAAACCGCCTTTTTCCTCTGCAACCACGGAGGATACCTGTGGGAGCCCGAGGAAACCGAGAATCCCAACCCTCGCGATCGCACCTACTGTCCGTGCAACTTGTGCAGTCCGCACCGGATGCCCCAACACAACGTGCCTCTGCACAACGAACTGCTCGCCATCAACACGTTTGAAATCCGCACGGACGACGGCAAGACCTTCAAATTGACTCCCGAACTGTGGGCCAACGCCTACCTAGACAAATTCGAACCCAAAGACTACCACCCTTTCGAAGTGGTGCACTTCCCTCAACACGAGGAAGCGTTCTCTAGAGACCTCACGGCCTGCGTCACCAAAAGCCCCGAAATCCTCAGTCTGATTCGTCAAATTCAGGCTTCGAGGGAGGAGTTCCTCCTCACGCGGGGTAAGGGCGTATACAAAGACCCCGACACCGGCGAGGTCCTCACTCCGCAGCCAGATCTCCAAGCTGGAGCAGCCCGGCGACAAGCTCTACCAACCGCTTACGCCGATCACGCCAGAGGAGCTGCGACGTCGGCAGAGCCTTCTCGAGCTCTACGGCCTACCAGCGTCGCAACCGCCGCCGGCGAAACCGAACACGGGGGTGCTCTTCAGCGCGCTATCGGCTCGGTCCAACCCTCCGTCGCAGGAGCAACTCCTCATGGCCCAGAGAATGGTCGACCTGAAGGCCAGGGCCTCGGAACCTCCGGAGCCCGAAATCTACAATCCCGAGGAGGCGACCGAGTCCGACGGCGAAACTCTAGGCAGCGAGGATACCGATACGGAAGAGGACCAGATGAGCACGATCTCCGAAGAGGAGGAGGAGGAGGAAGACGAGGCGTATTCCGCGGATCTGGCTGGGGAAGACAAGGAGAACAGCCCCCCTACGATTCCCCCCAAACGCAGCCGAAACGCATCCTCCGTCGCCCCGTCCCAGGCCCTGACGAGACCTCCCCTGCGTACCAACAACACCGCCAACACGAGCAGCACCGCCAGGAGGATCCGTCCGCAGCGCCTACCCGACCGAGCACCCCGAGGTAACTACCGGAGCTGGGCGCGCTATCGGGTGGCCATTTGTCAGGCGCTGCGGGATACGGTGTTCGATAGGGTTCAGGCAGCCCAAGTGCTGAAAAATACACGTCAGCTGTACGTGCCCGCCTCCGTGCTGGCTTACTACGCTAGAAAACTACTAGCTATGACCGACGACTCTGCTTTCACCCACAGCTGCGAGGGCTCGCAACGCTAGCCGAAAGCCTGCTGCGCCGCCTAAACCTAGAAAACCCGCTTCGGCCACTTCTTCGATTCTAGAAAAACCGATACCCCAGCACATCGCGGACCTTCGCGCCGAGGTTCTCGAGATCCTGCTCAAGATCGAACAGTACGCTCGAAAAAACCCCGAACGGCGAGTTTCCGTGCGCAATCGCACCCGGGAGAGCATCACTCGACAAGTGCATTACACCAGCTCTGAGGAAGCACTCACCAGGCTCAAGGCGGACGCGGAAAAAATCCTAGTCGCCTGGAGTGGCAGTGCCTAGACCGGGGACATTTATACTGTCGAAATGAACCTCTTGAACGCCGCACCCACCCCTTACGTGTGGAAATACAACCCCGTGACCGGTAAATGCGCCGGCGCCCAACAGAACTACGGCGCCACTATCGACTGGGTGTTGCCGGGCGGTAACAGTTTCGCTTACGCGGCCGATGAGATAAGGCGCCGATTCCCAGAACCGTCGGTCACGAGAGCAATTACCGCGCGCTTCGAGGCTGAGTCAGACCAACAGCCCTACGCGGGTCCGCACGAAACCAACATTATCACGGCAGATGTCGTGCGAAGCGGGCCGCCGCCCAGCGCGGTGTACCCATTTGACCCCAGCGGAGTCCAAAGGGTACAACTCTCCGGAGGCATGATGGGAGGTCGCACCGAGGGCAGGGTGCAATTATCGGGCGGACTGACCGAAGGTCGCATGCAATTAGCGGGCGGCGCAGCCGGGAAACTGCCGACGCGGGCGCGCCCTACTTTACGACCGCCTAGATGGTGCGGCACCACCCTGACCGGCAACGGTCTCCCGGCCGATTACCCCGAAATGACCCCGGACGCGTTCAAGTACTATCTACGTGTTCAAGGTCCCAGCCAGGAGGTGGACGAGCCCGGAGTCATGTCCCAGCGCCAATTCATGACCACGTTCCTCCCGGCCATGGTCCCCCACCCTTTCGACAGCGAATCTCCCGACGCCTTCCCGGCCTACTTCAGCAGCGTCTACAAGGGCACCAACGCTTTCGAACCGGTGTTCTGGCAGGGTTAAGGGCGGTACTTACGCGCTGCTGACGGTAATCGGTCTCGGAATAAAGCTCTTGCATTTCCGAAGCGGAAAATGGCTCCTTTCTGGTCATTACGACCCGCTCGCCGCCTTCGCTGTGTACGCGCGCTCTAAACTTGCGTCTCAACCAAAACACGAAACCAGGGTTAAGCGGTTCGTCGAACCGTAACATAGCCTGATCGTTTATTTTTAACCAATATCTTCTAGGCTCCGCCATGTCGGCCCTAATCGCCTCCGCAGCCGATACCGTCTCCGCCAGCGGAAAAAAACGACCCCGCAGGGCCCTATCCGAACCTAGCCGGTACCTTTCGGAGGGCGACGAGCGTCGAAAACCCAAACGCGCGCGACCGGCCACCCGCGCGAATGGTCCCCTCCTCGATCTGGTGTATCCATTTGACTTCAATGCGGGGGGAGGAGGTGGCAGCGGTGGCGGCGGTGGGGGAGGTGGAGGTCAGCAGATCGCGGTCGACCCCGATGGGCCGCTCGAACTCACTGGTGACCTACTGACCCTCAACACCAAAACGCCCATTTACGTCAGCGATCGAGCGGTCAGTCTGCTCATCGATGACAATACTTTGGCCACTAAGCAAGCCAACGGGGCGCTCATGGTCAAAACCGCGGCCCCTCTGAACTCGGGCACTGGTGGAGGCGTCACGCTAGGCTTCGACCCTCGCACCATGGCGCTAGATTCCGTCACCGGGGTGCTCAAAGTGCTCGTCGACTCACAGGGACCTCTACAAGCCGACACGGGAGGCATCACTCTCCAGTTCGACACTCAAGACTTCGTTGTCAACAATGGCGTCTTAGCGCTAGCCTCCTCGGTCGGTCCGACCTATCTGAGCCCCTTTGCGACCTACGAAGTCACGCCCGTCTTGGGAATATCGCAGAGGAACGGCAACGTAAAAAGCAAGGGCTTGCAAAACTGGTCCATAGGCTATTACATCTACATGGTGAGCTCAGCCGGGCTAGTCAACGGACTCATCACTCTGGAGCTAGCCCATGACCTCACAGGCGCGAGCGGAGAAAACAGCCTGACTAGCGGTCTCAACTTTACCTTTGTGCTCAGCCCCATGTACCCGATAGAAACAGAGGTGAATTTGTCCCTCATCGTGCCGCCCACGGTCTCGCCGACCAATCAAAACCACGTGTTTGTGCCCAATAGCAACCAGAGCGACGTGGGCTATCTCGGGCTGCCGCCTCATACCAGGGACAATTGGTACGTGCCCATCGACTCGCCCGGCCTGCGGCTCGTCTCTTTCATGCCCACCGCCACCGGAAACGAGAAATTCGGACAGGGCACGTTGGGATACTGCGCCGCCACCATCCAGAACACGTCCAGCGGAACCACGCCGTCGGATGCGATAGCCTTCACTGTCTCGCTGCCGCAGACCTCCGGCTCCAACTGGTTTGACCAGAACGCGCCCGACACTGTGGTGACGACCGGTCCTATCCCTTTTTCCTATCAGGGTTACGTCTACTCCCCCAACGGGAACAATGCTCCGGGCCCCTAAAAGAAGACATTCCGAAAACGGGAAGCCCGAGACCGAAGCGGGACCTTCCCCGGCTCCAATCAAGCGCGCCAAACGCATGGTGAGAGCATCCCAGCTTGACCTGGTTTATCCTTTCGATTACGTGGCCGACCCCGTCGGAGGGCTCAACCCGCCTTTTTTGGGAGGCTCAGGACCCCTAGTGGACCAGGGCGGACAGCTTACGCTCAACGTCACCGATCCCATCATCATCAAGAACAGATCGGTGGACTTGGCCCACGACCCCAGTCTCGATGTCAACGCCCAAGGTCAACTGGCGGTGGCCGTTGACCCCGAAGGGGCCCTGGACATCACCCCCGATGGACTGGACGTCAAGGTCGACGGAGTGACCGTAATGGTCAACGATGACTGGGAACTGGCCGTAAAAGTCGACCCGTCCGGCGGATTGGATTCCACCGCGGGTGGACTGGGGGTCAGCGTGGACGACACCTTGCTCGTGGATCAGGGAGAACTGGGCGTACACCTCAACCAACAAGGACCCATCACTGCCGATAGCAGTGGTATCGACCTCGAGATCAATCCTAACATGTTCACGGTCAACACCTCGACCGGAAGCGGAGTGCTGGAACTCAACCTAAAAGCGCAGGGAGGCATCCAAGCCGACAGTTCGGGAGTGGGCGTTTCCGTGGATGAAAGCCTACAGATTGTCAACAACACTCTGGAAGTGAAACCGGATCCCAGCGGACCGCTTACGGTCTCCGCCAATGGCCTAGGGCTGAAGTACGACACTAATACCCTAGCGGTGACCGCGGGCGCTTTAACCGTGGTCGGAGGGGGGAGCGTCTCCACACCCATCGCTACTTTTGTCTCGGGAAGTCCCAGCCTCAACACCTACAATGCCACGACCGTCAATTCCAGCGCGAACGCCTTCTCTTGCGCCTACTACCTTCAACAGTGGAACATACAGGGGCTCCTTGTTACCTCCCTCTACTTGAAATTGGACAGCGCCACCATGGGGAATCGCCCTGGGGACCTCAACTCCGCCAATGCCAAATGGTTCACCTTTTGGGTGTCCGCCTATCTCCAGCAATGCAACCCCTCCGGGATTCAAGCGGGAACGGTCAGCCCCTCCACCGCCACCCTCACGGACTTTGAACCCATGGCCAATAGGAGCGTGACCAGCCCATGGACGTACTCGGCCAATGGATACTATGAACCATCCATCGGGGAATTCCAAGTGTTCAGCCCGGTGGTAACAGGTGCCTGGAACCCGGGAAACATAGGGATCCGCGTCCTCCCCGTGCCGGTTTCGGCCTCCGGAGAGCGATACACCCTTCTATGCTATAGTCTGCAGTGCACGAACGCGAGCATTTTTAATCCAAACAACAGCGGAACCATGATCGTGGGACCCGTGCTCTACAGCTGTCCAGCGGCCTCCCTCCCGTAAGCGCGCCCTCCCCACCGCGTGTCAAATAAAGAGTCATGAACGTTGATTGCTTTTATTGATCGTCCATTTGTCCGAAAGCTTCTCTCCTTTGTTCCCGCGTCCAATCATACAACAGATTTACGCTTTCTAAAAACCAATCGGGTGCGTAAAAGCGAGTAATGTTGTGTTCCACACTGATTTCCATTTTGCGCAAGTAGTGACAGGGGAGCGCCCCCAAGGCTCCAAGCTGGGATACTACCATCAGGAATTCGGCCCGTCTGTCGATAGGGTAAAGAGGTACATGAATCATCATGCCCACCACGCCCTGCAGGTGGTGGACTACCATGGGTCCAAAGGAGGAAAACATGGCACACGCCCACGCGCACACAAAATAGTTCCCGGCTACCCCGTAGTCCTCCGAAAGTCCCTCGCACAGCTTATCCCCGTACCTACAGTACACCCCGGTTCCCACAGACAACCCCAAATTGCGCAAACCGTAATTGTGACACACACAGTTTTTGACCACCACCCTCATTCTCTCCATGCAAGCAAGCACCATGTTTTCATGCGGATTGGCTGCGGCCTCCGCCGCTGCGAGCTCCTCCTCCTCTTCGGCAAGGGGGGCCTCCTCCATCGGTTCGGGTGATGCGTCACGCTCCTCCATCTCTAAACCTGGGTCTCGTAGGGCGAAATGGAAGTGTACACGTGATCGTAAGGGTCCTCGAGTTCAGAGTATGGGTTGACAGGCACAGGCGGCAGCGGTCGCGACGCGCCTAACCCGATGCGCAAACTAGAGTACAGCGGATTGGCTGACAGTACCCACCTACCCGATCCCCGCCTTCTGAGGGACCGGACCTCTGGGGGCGTGCAACGGGCCGTCGGAACCTCACAAGTCCACTCCCTGATAATAAAGTACAGCACAATCAACACCATTAAACTGCTGGTGAGAAACACGGTTATGACATAGAGCAACAGACTGACGGAGCGTTCCACGAAAAGAAAACTGACGGAAATGCAGGTAAAGTAGAGACCGACCGTCAGAAAGCCGATCAGCAGCAGCGGGAGATATCTCCGTCGGCACTCCTATTTAAAAAACGGAGATTTGCGATTGTGAGTCACGCGAGCGCGTCTGCGGTCAAAGGCCAACTTCAAACAAGCCATTTGCGCCAGATAGAACAAGTTCATAGGAGACTTGACCTCGCACCGATCACACCCTTTCTCGCAATCACAGAAGGGACCGTGCACATGCATAAACCACTCTTCCATGTCGCTATAGGTGGCGCGCCTGTGAGCTGGTAAATAGCACACCCCGCGAAACACGATTTCCCTGTACGGTAATGGCACAAAATCGACCAGCATGCTTTTCGTGCATATCGATTGAGTGATACAGCTACCATTACCTGGAGGACAGACCAGAGTCATCCTCACATTGGTTTTGCAACATTCTAGGTAATTGATGGGCACCTCCATAAAGGGAACACAGCGCGGAACTTTAGAGGTGGAAATCTCAAAACACATGGTGCGCACGGCCATCTTGCAGCATGCCATGCTCCCCAAAACCAATCCGCTCGGTACGAAAATAAAATCGGTCTTCCTTTTCCTTTCCATCCACCTGACCATGGGAGCCCTTATCTCAGGCGGAACGTTAAAAAGACGTCGGGAAATGAACTGCAAAAATTCCCGCTTGTCAGAAGCGGTGAGCGGAATAGCGGTCTGAAAGCGTATATCGCAGTTCACCACTAAGTGAACTCCGGACCGCAGGCGAAGATAGCGTGAACACCTACATCCCACCGCTACGTAAATCGATGAATGTATGTAGCGCACAAACTCATCTCCGTTAAAAGTGTTCAGATCCAATATCGCCAAAGTCAGGTGATATTCTATCATCCTCTCAATCTCTTCCTGATGGAGCACGATAGGCTCCCGAGGTGGGGGTACCAATACACTGAACGAACAACACCCGCATTCCATTTCAAAACCGCATCCGCGAACCAGGGCCAGAACGTCCTAGAAATAGTAATAAGCTCTACTCAGAACTCTCAACAGAATCACCCTCCCCGAACCCGAAACCGAACTCACCGATTCAGCACACAGAGGCATGATCTTACAGCAGACCGAGGATTTTATTTACAGCACGGTCTAGAAGAACAAATAAATAACAGCAGGTCATTTCGTGGCTCAGGCACAGCCTATCGGAATGCGCGACCTAATATCACGGGATAACATAAGAATCAGGGGTGGCCCGTATACTAATCCCGTCACTGACGACACCCACAATCACGCAATGCATAGAGTACGGCTCAGAGGCTCCTTCTCGAGTGGAGTACTAGCCGGGCGAATAAACTCCGATCCGAACATCCGCCATATAAACACAGCTTTCGTAAAATATTAACAGACACTAACTTCCTCATTGACCCGCTTAATTGCCGTGTCAGCAGGTGGCTGACAATGAGTCATCAATGAGTCATCGCTAAGTCAACAATGGAACTTTCCACTTGCTAACAAAGCGAAACCAAAAGTTAATCATTAACGCCACACCCATGATGAGTCATCGCCAGTAAACCTTAAAAGGACGGCTCCAGCCCGCAGCGAAAAGCAACCTTACTTCCAACACGAGGGCTCTGCGGAACCATGGCCGAAGAGTGGCTCGACCTTTTCCACCCCTCCACTTCGCCGAATCCAGAAGGAGAAGGTGAGGACATGTCCCTCGAGACCGAGTGCCATGCCCCTCTTCAATATATTTCCATGCTGTCTTTTGATGACCTCCTGGCGGCTGCCGGTCCCCCGGACTACTCTCCGGAAGAGAACCAGGAAACACCGCCGATCGAAACCATAGAGGTAGGAGACATCATGGCCGAACTCGGTATTCCGATAGAGGGACCTCCGACCAGCCCTTCCGACTCTTCCTCCAGTTTGGATTCAGTACTTTTCTCCGGTGTCGACTTGTATGACTTAGACTATACCATTGTCTTTTCCCGACTCCGTGAGTTTTGGCAATCGCACGGCGCATACTTGAAAACCGTAGCTTCGCTCGAGTGCATGCAAAACGACAGGAAATTTCAGGAAGCATACTGCTCACTGGTGAGAATGCACGCCGTTTCCGAAGATGCCAAAGAGCATCTCAATGAACTCTTACTAGACGAAACCAACTACCAACATTGCGAACCCCTCAATGACATGTTGGACTTGGGATTCCGGTGGCTCAATGACCTAAAAGGAGGAATAATGGAGTGGTGCATGGACACTGCCCTGGATCGCGCATCAAAAGTCATGCCTCTGACTGACTATCAACCACAATAAATAAATTTTACATTAAAAACTTTCTGAGTAAGATTTTTCTAACCTGAAAAATTCTAAGTGCGGTTAATCATTAATCAAGTTAAATATTAAACTCTAGTTAATTATTAAACTCTAGTTAAATATTAAACGCTAGTTAAATATTAAGCTGAAGTTAATGATTCATCCGAGTTAATGGATAACCTTGAGTCAATGATTAACTCTGGTTAATATGTAACTCGGCTAATGATTAACATGGGTTAACCATTAACATGGTTTAACCATTAACTATAGTTAATAAATAACTCTAAGTTAATAAGTAGCTAGTGACGTACGATTGACGTCACGGTGACGTCGGTGTTGCCATGGAGATGTAACCATGGTGATGTTAAACATTAAACTGCTGACACCAGTGGAATTTTCCATGTTAACCATTAACATGGACCTTGTCCTGTTTGTTTATTCACCATGGCAACATACCATATATGGACATCCGACTCCGCCTCCTCCGTTATACATTAACGATGGCGTGATAGGCGGAGCTCTCTCCCATTGGCTCTCAATGATGTCATGTAGTTACACATTAGCCCGTTCAACCTATATAGGTAGACCAGGTAGGCAGGTTCAGACAGACAGACCGGGGACCAGCAGACTGAACGGAGCTCTCCACTAAACCGGTAGGCCTCTATATTGAATCGATGAATAAATACCGAATCAACTCAATATTATGATTTTCCATTGAAATTAATGGTGATTTTCTTCAATCAAACTCCCACCCCCCTTGGCACCCCCCTGTACACCCCCCTGTACAGGCGACCACCCCCTATGATCACCCCCCTGTACACGCGACCACCCCCTATGATCACCCCCCTGTACAGCCGACCACCCCCCATGACCACCCCCCTGTACCATTACAGCCAATGGGATCCCATCCATTGACATCACATGATCCCCGCTGGCCCTATGAGGTGGCTACCATATCCTTCACCCTATTGGATCCCATGCCGAGGGGCGGAGAAGATGGGAGGCGCCCGTACCTCGACAACCAATTGGCTGAGGCCCTTCAGTTCAGTCCCGCCCTCACTCCCGACCAATCCAATGCATTGGAGTTCACCACGTGGCTCGTGAAGGGGCGGAGACTCCTCCATAAGGGAAAGCAGTACCGCCTCTACAATATGGCGGCCCGGATATGCAGTATCCACCAATGGGAGAGAAGTGAGGCCCAGCTGACCATGGAGGCCGTAGCCAATGGGCTGTGGGATCTGCCGGATGAAATACTCGGGAGCCCCCTACTGCACAATACTGGTATACACACCTGGGGCTGGGGGGTCCCCGTCACTACCGAGATCAGCCTGAAGATGGTGCTAAAGACCCTCCGTGTCAATACTCCATTCAACCGCCAGGGGGAGATGCCTATACCGGTATCCAAGGAGGTCCATGTAGAAGCCCCCCAACATTTTGAAGATATGCTCCAGGGGGTCCTGACGACCACCGATCTAAAAAAGCATATTCCTAGACCCATCTTTTCCCGATTTTTTAACGAAAAACCCTCGGTTTGGGCCTATAAGACTTTCAAATATTCGGCCGGTGAAGAAAAATGGCGAGTGGTGGTCCCTACCGAGGGTCCCTATGGGGGTCCTAAGAACCCTGTTTCTCTGCAAAACCTCGCGAAAATGGGTGTGTTGGAAAATTGTCTAAAAATGAAAAGGGCGGGGCTACGGTTCATGCCATATTAATAAACCAATCAGAAAACAGAAATACGACTCCTCCTCTTTGTGGGCGGTCCTGGGGAACACCAATAGAAATAGAGACTCCGCCTATGAGGCGGAGACTTAGTTACTGAATAACTTTTCGGACTTAGAAAAATTTTCACCTGCTTAATCATTTACCAATGGGCCTACGTCACTATGCTCCGCCTTTAACTCCGCCTATAGCTCCACCTCTCTCTCCGCCCCGATGGACTTTGTCCTTTTGAGTCACATGACTGCTACGTCAGAGGAGGAGGAGCTTCGGACTTAGAAAATTTTTCGCTCTATCAATCATTAACTTGACGATGGACTTTGAACCCCACGTATGAGAGGGGGAGGAGCTAACGTTAATCTTCAACTCGGACTTTGCCCAGAGGCGGAGCTTCGGACTTAGAAAATTTTCCACTCTATCAATCATTAACTGAGCAACGGACTTTGGCACTGACGTGCGCAAAGGGGAGGTTCTAAAGTTAAACTTTAACTCGAACTTTGTCCGGAGGCGGAGCTCCGGACTTAGAAAAATTTTTCACCGCTATAATCATTAACCAACTGAATCACATGACACAAAGGAAGAGACTGTAATCAAATTTAATTATTAACAACTCAGTCAACATTTACATCATCAGCGGTACGAAGTCCAGTACATACACGCCACGCCCCAAGACATTGAAATGCTTCCTCCGTCACGCCCCAGCGCCCCATGCGGTACTGCTCGGGACACCACTGATTATCATAGAGCTTCAACCACACCTGGCACGGAGAGCCGGTATCTCTATAAAAAACCAAATGCAGGAACCGTCGCATATCACTCCGACACTTCTCCAACTTTCCCTTAGTCTGATCGGAACAATGGATACAAATTAACAAAGTTGCATGACATTCGCACACGGAAAACTCTTGCAACCCGAACATGTACTTCAGAGGACTGCATTCGGGACTGTACTTCTCAACAAGTTTAGTCCATATGAACTTCAATCCGCGATGAACATATCTGAAAACCTCAGGCCTCTGACACCATTCCGGAACTGCCGCTCCGAACACAAGGTACATTGTCATCTGAAATTTAAACCATTTATTCATACACGCACTCGTAAATACATCCCTCTCTACTGCCAACCGCTTCTGATGTTATGCAATATTCACCCAAGCGGTACTCTTGCGCGTATCCCTCGTTACGCAAAAGGCATACACCCTTAATCCACTTCTCACTGTCATATGGATATTTCCCAAAAAACAAACACTTAACAGCCAAACCAACCCCCTCCAGATACGCGCTTCCAAAATCCGAACAAGGCAAAAAACAATCGATACAAAATCCTCCATCTTGCAAAGCATAGAGATAAAAGAAACCCGTTACTAGTCCAGGAAACACTTTTCCTAAATCCAATTTCTCCCAAGCATTAATTATATCACGCCGAAAATCGTGAAACATAGCCAGGTTCAATTCCCATAAGGGCACGCGCAACATTAAGTAACCCAACATTACCCACCGCACACTGGGCTGTCAAATGAGCGCTCTGCAGTCCTCCAAACTCTGTAGTACTTATACAGTCTACCTCTTTAATCATTAACCATAGACGTCATGGAAAATTTCCAACCCCACCCAGAATGAATCACCGGTCAAAGGTCACTCCCTCATTACCTAGATAAGGATCACAAAGTACTTCCTCATTACCTAGATAAGGTATCACAAGGTACATGAGTCACCCATAATGATACTGAATCAGCGGTTTCGGTCACGTCAGCATCAGACAATCATTAACCAGAAATCCCTATAAACTTTCCGCAGACTCGCACCACGGTATTCCATCCGAAGACTGATCCAGTCTGAAGATACTCTTCTCTTCGGTGACCAGTCCTGAGGAAGAAACACCATGCGGGTAAGCATCATTCATCCATTACCCCTTTTACTCTCTCACCTAACCTCGCTAACATAAATCTCCTTACCTCTCCATCCACAGCTGTTAGCCCTACTAACCCTCCTCACTGCATGGTTCCTAAATGGGGATGCTGCCGTCAACTCCATACAGGACAAACCCACGGTCCCTACCAAAGGACCCATGGCCAACTTCAGCCCGGTTCGCCATGAAGGTCACATCAATTACTTTTGGTATGGGCAACACGGCATGGCACCCCCGAGAATCCATGGCCCTCTCCATGACAATGACATGATCTACTGGAGACTCCGTGACCGTGGATTCCTGAGAGGCGGAAGGGAAAAGAACCTGATCCTATTAGTCCATGGATGGCACGGTCTCCACCGCACCTTTGATATCTTCTTCAAATTCCTCCGCTTCCACCAGAAGATGACCCCAGACGTAGGCGTGCTGTTAGTCGATTGGGGGGTACAAGGCGCCGATAACCTCATTCTAGGAGATGCCGCTTACCACGCCGTCACTATCAATATCGACGGATTGCTCAAGAACATAAACCGCACCAACTTACACTGCATAGGACACTCCTTGGGGGCTCATGCATGCGGTGCAATTTGTCGAAGATTCAACCAGCTCCAAAATAGAAAATGTACTAGAATTGTTGGACTCGACCCAGCAGGACCTCTCTTCAAAACCAACTCTCCCTATCCTTACCTCACCAAAGCCCGTCTGTCTAAAAAAGATGCTGACTATGTAGCTCTCTTTATGACGAACCGCCGGATGATGGGACTCCACGAATTAGAAGGGGATGAGTACATTACCCCTTACATAGATGGCACCTATTTGAATCACTGTCCCTTCATTGGCAAATGGACAGGCACTATCACTGCCGAAAATTACCAAGGAAGAAAGGTCACGGAATACATCGACTTAGGTACGGTGGCCAAATCGGGTGTAATCCCACACACCATGGATGCATGCTCACACCTCATGGCTCCTGTTCTTTTCATGGTGTCCCTAGACACCCGTCAAGGCCTACCTGCATTCCGGTATGCTGAGAACCCTCCCCAAGATCAAGGTGCCATGCATACGGTTTGGAATGGGTACACCATAGGGAAAGACTACCAGTATCCAGCCTATTTCAAACACGAAACTATCTGGCTTAGTACACTCACCACGGATGCAAACCAGCTCTCACCCTTCGAATTCCAACACGAAGATTCCATAGATCCCTCTTTCATGGCAATGGCAATTAGCGACAAGGGATGCATCTCGGCCGGCTCCCATCTGAGCTACCACTACAGTGTCATCCCTTACGGAAACAAATACGATTTAGTAACATCCTTCAGCGCACTCTCCCCCGGAATGGCAGATACGCACTTCCTCGAGGTCTACATGAACTACAAACACTGTCCCGTCTATCTAGCCCGATTTCTGATTCCCAAACCTTACCAACAGCAGTTACCTAGACCCACCACAGCCGGCCTCTCGTCCGAAATGTTAAGTTGCAGGAAACAAACCACTTATACGTGGAGCTGCTACAGAACATGGAAGCAAGCTGTCCTACCCGTGTACCGCCAGCAACTCGATCTTACGGGTGACGGAAGGCACAACATCCAAGTCCCTCCCAAACATGGATGCCTGAAGGAACAATCCAACTTTACCGACATGTTCCGTACATACATGGGCGCATATGAAGTCTTGACTGATCAGACTGTTACAGTAACCAGCTTGCCATCGCCGTTCGAACTCATCCGTATTGCTCTGAGAGATCCTGCCTCGCCCACCATTCAAAACATCATGACCTACTGGGACATGTGCGATCCTGTAGCTAGCACTTGTAGCTTCACAGTAAATCGAGCTACGAGAACTCTCAACATAACCTGCCCCGACCCGAAGACCTACTGGATCTCCTTCTTTTACCAATGGGAAGAAGTCTTGCTCAAGATTAATGTCCATCCTAAACCCACTACCACTACCACCACTACAACCACCACTACCACTACCCCTACAACCACTACTCCCACTACTACAACCACTACAACTACTCCCACCACTACAACCACTACTCCCACCACTACAACCACTACAACTACTCCCACCACTACAACCACTACTCCCACCACCACTACCACTACTCCCACTACGACCACGTCCTCCACCGAATCAATCACAGAACCGAGCTCCGCTTGTGATGAAGAAGAAGATGAAGATTGTTGGTTCGAAAAATATCGCGATCAGATAGAAGTTCCTCAAAAGGTACAACTCCCGTTCAAAGTAGTGAACAATGAAACCTCAGAACCAACTACTGCTGCTACTACTCCTTCCAGCACTGCCATCATAGAGGAAGAAGAAAACAGCACGGAACCGATCGAAGAAAGCAACAGCAGAGCATCCACACCTCCCCCTCTTCAACTCACCGTATCCCCAGGTACTAACCCCCCTCTTCAAGAATTCCTCTGGGCGGAACCATCATCTAAAGATTCCCTCCACAAGGACCAAGACTCCACGGTCACCATTCCTGTCACCATTGGACTCTTAGCCCTAGTCTGTCTCAGTGTTATCATTGCCGTATTCATTGCCCTTAGAAGGAGAGGGAGAGGTCCAGGGCCAACCTTTATTGTTGTTCCTGGAACAGGCAATAATACTGTATACCAGGAAACTACTGAAATGTTGTAAAATTTATAACGCTATAAAAGTGCGTGACTACAATAAAGATAAGAGCATGAATCAACTCG**CGTTACATAACTTACGGTAAATGGCCCGCCTGGCTGACCGCCCAACGACCCCCGCCCATTGACGTCAATAATGACGTATGTTCCCATAGTAACGCCAATAGGGACTTTCCATTGACGTCAATGGGTGGAGTATTTACGGTAAACTGCCCACTTGGCAGTACATCAAGTGTATCATATGCCAAGTACGCCCCCTATTGACGTCAATGACGGTAAATGGCCCGCCTGGCATTATGCCCAGTACATGACCTTATGGGACTTTCCTACTTGGCAGTACATCTACGTATTAGTCATCGCTATTACCATGGTGATGCGGTTTTGGCAGTACATCAATGGGCGTGGATAGCGGTTTGACTCACGGGGATTTCCAAGTCTCCACCCCATTGACGTCAATGGGAGTTTGTTTTGGCACCAAAATCAACGGGACTTTCCAAAATGTCGTAACAACTCCGCCCCATTGACGCAAATGGGCGGTAGGCGTGTACGGTGGGAGGTCTATATAAGCAGAGCTGGTTTAGTGAACCGTCAGATCCGCTAGCATGGTGAGCGGCTGGCGGCTGTTCAAGAAGATTAGCTTAGCGGCCGCGACTCTAGATCATAATCAGCCATACCACATTTGTAGAGGTTTTACTTGCTTTAAAAAACCTCCCACACCTCCCCCTGAACCTGAAACATAAAATGAATGCAATTGTTGTTGTTAACTTGTTTATTGCAGCTTATAATGGTTACAAATAAAGCAATAGCATCACAAATTTCACAAATAAAGCATTTTTTTCACTGCATTCTAGTTGTGGTTTGTCCAAACTCATCAATGTATCTTAAGGCGTAAATTGTAAGCGTTAATATTTTGTTAAAATTCGCGTTAAATTTTTGTTAAATCAGCTCATTTTTTAACCAATAGGCCGAAATCGGCAAAATCCCTTATAAATCAAAAGAATAGACCGAGATAGGGTTGAGTGTTGTTCCAGTTTGGAACAAGAGTCCACTATTAAAGAACGTGGACTCCAACGTCAAAGGGCGAAAAACCGTCTATCAGGGCGATGGCCCACTACGTGAACCATCACCCTAATCAAGTTTTTTGGGGTCGAGGTGCCGTAAAGCACTAAATCGGAACCCTAAAGGGAGCCCCCGATTTAGAGCTTGACGGGGAAAGCCGGCGAACGTGGCGAGAAAGGAAGGGAAGAAAGCGAAAGGAGCGGGCGCTAGGGCGCTGGCAAGTGTAGCGGTCACGCTGCGCGTAACCACCACACCCGCCGCGCTTAATGCGCCGCTACAGGGCGCGTCAGGTGGCACTTTTCGGGGAAATGTGCGCGGAACCCCTATTTGTTTATTTTTCTAAATACATTCAAATATGTATCCGCTCATGAGACAATAACCCTGATAAATGCTTCAATAATATTGAAAAAGGAAGAGTCCTGAGGCGGAAAGAACCAGCTGTGGAATGTGTGTCAGTTAGGGTGTGGAAAGTCCCCAGGCTCCCCAGCAGGCAGAAGTATGCAAAGCATGCATCTCAATTAGTCAGCAACCAGGTGTGGAAAGTCCCCAGGCTCCCCAGCAGGCAGAAGTATGCAAAGCATGCATCTCAATTAGTCAGCAACCATAGTCCCGCCCCTAACTCCGCCCATCCCGCCCCTAACTCCGCCCAGTTCCGCCCATTCTCCGCCCCAT**GTGTCCGCCCATTCCTTTCTCTATATATTCTGACATGAACAGATTTCAGACAGAACACGACATGGGAGGCGAGAAGAGCTCCGTAAAGCTAGACAGGTTCCCCTACTGGGGTACCCTAGAAGAAATAGATAGGTATGCTAAGGCTAATCGCGGAACAGTGACCCCCATCGGGTCCGGCAAACACTTTCTAGTTATAGGAGATCTGGAAGGTACCTTACATGCGGGTCAACATTTAAAGGAATACTGCGAAGTGCTGTATCTGCCTTCCCCAAAAAGAATGACCGTCATTGGCATAGTGGACAACGTCATCTCATTCGCGGATGGATTGCAAGTAGTCATTTTGGTGGCGGAAGATAAAACCGTCTATGGCTACGAAGAAGACACTCTCCATAAATTAGCAACCACCATACCAGAATTCTTTCGTATCGGAATGCAGAACTTTGGAACCGAAGTATTTCACTGCGGTTCCCACATCCCCCCATTGGTAAGTGCAGATCCCACACCCTCTCATTACTTACCTGATAGATACTAACACACTATATTCCAGTCCGAGGAGGAGCGTCAGCGTGATCCCGAGATAAGGCGGCTCCGAGAAGAAGCTCGAAAGTTCATATCAGCCGGCGAAAGAAAAACAGACTAACCAACCGCAATCCGATCCGAACATAGCCACGCAATGGTGTGCGGATCCACTTAAAATAGATTACGCGTATTACCAGAAATAAACTGATTGAAATGAGAGGCAAGAGCTGTGTCATTATTTCGCGTTCGTTCGCAAATACGGAAGTCCATCACGGATATCCGTAATCGTCATTTGGGTGGAGACCATGAGTCATTGATGACTCACTTAAACGGTTTCGGTTTCGGCTATCACGACGCGCTGCGCGCGGCGGTTGTAAGTGTGTCAAAAGACGCGGTTATATAAGATGATGATG

Underlining indicates HiBiT-expressing cassette sequence, and the yellow part is the full junction sequences on both sides of the insert fragment.
